# Strategic complexity and cognitive skills affect brain response in interactive decision-making

**DOI:** 10.1038/s41598-022-17951-0

**Published:** 2022-09-23

**Authors:** Carlo Reverberi, Doris Pischedda, Marco Mantovani, John-Dylan Haynes, Aldo Rustichini

**Affiliations:** 1 Department of Psychology, Università Milano-Bicocca, Milan, Italy; 2 Milan Center for Neuroscience, Università Milano-Bicocca, Milan, Italy; 3 Charité-Universitätsmedizin Berlin, Corporate Member of Freie Universität Berlin and Humboldt-Universität zu Berlin, Bernstein Center for Computational Neuroscience Berlin, Berlin, Germany; 4 Charité-Universitätsmedizin Berlin, Corporate Member of Freie Universität Berlin and Humboldt-Universität zu Berlin, Berlin Center for Advanced Neuroimaging, Berlin, Germany; 5 Department of Economics, Università Milano-Bicocca, Milan, Italy; 6 Department of Economics, University of Minnesota, Minneapolis, MN US

**Keywords:** Cognitive control, Decision, Intelligence, Problem solving, Neuroscience, Cooperation

## Abstract

Deciding the best action in social settings requires decision-makers to consider their and others’ preferences, since the outcome depends on the actions of both. Numerous empirical investigations have demonstrated variability of behavior across individuals in strategic situations. While prosocial, moral, and emotional factors have been intensively investigated to explain this diversity, neuro-cognitive determinants of strategic decision-making and their relation with intelligence remain mostly unknown. This study presents a new model of the process of strategic decision-making in repeated interactions, first providing a precise measure of the environment’s complexity, and then analyzing how this complexity affects subjects’ performance and neural response. The results confirm the theoretical predictions of the model. The frequency of deviations from optimal behavior is explained by a combination of higher complexity of the strategic environment and cognitive skills of the individuals. Brain response correlates with strategic complexity, but only in the subgroups with higher cognitive skills. Furthermore, neural effects were only observed in a fronto-parietal network typically involved in single-agent tasks (the Multiple Demand Network), thus suggesting that neural processes dealing with cognitively demanding individual tasks also have a central role in interactive decision-making. Our findings contribute to understanding how cognitive factors shape strategic decision-making and may provide the neural pathway of the reported association between strategic sophistication and fluid intelligence.

## Introduction

Deciding the best course of action in a social setting can be a demanding problem. It requires the decision-makers to integrate their preferences and those of others since the outcome depends on the choices of both. Traditionally, game theory analyzes these interactive situations by assuming individuals’ full rationality, thus providing an essential but limited benchmark to analyze real social interactions.

Different games model diverse social interactions. Of particular interest are *repeated games* in which two players play successive instances of the same game for many, possibly infinite, periods. In these games, one’s future actions can depend on the other’s past actions. The repeated Prisoner’s Dilemma (*PD*) reproduces situations characterized by a conflict between an action (defection) yielding an immediate larger reward at the partner’s expense and another action (cooperation) yielding instead a larger cumulative reward for the two players in the long run. The repeated Battle of the Sexes (*BoS*) reproduces situations where two players have incentives to coordinate on one of two outcomes, but each player prefers to coordinate on an outcome different from the one preferred by the other player. Repeated games have multiple equilibria, and standard equilibrium analysis is silent on which outcome we should expect to emerge^1^. Nevertheless, understanding behavior in these strategic environments is essential as they represent the archetypes for studying cooperation (*PD*) and coordination (*BoS*) in repeated interactions.

Empirical investigations show that individuals’ behavior in strategic situations is heterogeneous both in the laboratory and in the field^2^. In *PD*, for example, some people tend to cooperate more often than others, although they face an identical strategic situation. Significant efforts have been devoted to uncovering possible individual determinants of these differences. Two main areas have been considered: the personality of the decision-makers (assessed with, e.g., the Big Five inventory or prosociality index) and their cognitive abilities (e.g., fluid intelligence).

It has recently been shown that the average fluid intelligence of the interacting decision-makers has a central role in predicting behavior in games^3–5^. Groups with higher fluid intelligence tend to cooperate more in the repeated *PD*, reaching almost 100% cooperation rates after a few iterations, compared to 40% in the lower intelligence group. This disparity produces a substantial difference in the final cumulative earnings^5^. However, while prosocial, moral, and socio-emotional motivations for cooperation have been extensively studied for at least three decades^2,6–12^, the cognitive and biological pathways connecting fluid intelligence to strategic decisions remain mostly unknown. Here, we fill this gap by modeling the strategic situation and the players’ reasoning constraints^13^. We additionally investigate the neural correlates of strategic decision-making and their relation to inter-subject variability.

For this purpose, we devised a task reproducing essential aspects of the strategic environment a player would face in an interactive task with a human partner^14–16^. We replaced the human opponent with several pre-programmed strategies (i.e., automata). Artificial opponents allowed us to isolate the cognitive component from the social and moral ones^17^. Participants faced a strategic environment in which two main dimensions changed pseudo-randomly: the active game and the interaction partner. The game was either a *PD* or *BoS* (see Methods for details). The artificial opponents represented strategies typically used by human subjects in these games. The active game and the opponent’s identity were always known to the participants, who could thus adjust their behavior according to the specific strategic situation. Controlling the type and variability of the opponent’s responses allowed us to ensure similar decision environments for all subjects.

To study how the complexity of a strategic situation affects behavior, we need a reliable measure of complexity. To construct it, we developed a game-theoretic model of the strategic environments and the participants’ decision-making. Based on this model, we provide a precise measure of both the environment *strategic complexity* and the subjects’ *performance*. The method we follow is to model strategic behavior as implemented by automata, that is, by algorithmic representations of the rules followed by the players. An automaton consists of a set of internal states, a function assigning an action to every internal state, and a transition function mapping the current internal state and the action of all players to the next internal state. When a new instance of a game is played, the transition function considers the current state and the actions chosen by the players, then decides the new internal state. An action is then chosen as a function of this state, and the automaton proceeds to the next stage. The complexity of the strategic environment results from the size of the opponent's transition function and the nature of the current stage game. Our fundamental assumption is that a more complex strategic environment will be more cognitively taxing^18–20^. This investigation is part of a recent line of research on the neuro-computational modeling of social behavior^9–12,21^. Our method is novel in integrating the theory of strategic behavior in repeated games and the cognitive processes underlying it.

Our model provides us with performance measures, for example, the loss in total future payments incurred by choosing one action over another. By exploiting these measures, we can formulate predictions at the behavioral and neural levels. Behaviorally, the theory predicts that the participants’ performance will inversely correlate with the environment complexity, as determined by the game and the opponent’s strategy. Furthermore, we expect participants with lower fluid intelligence to fail more frequently in more complex strategic environments; instead, inter-individual differences should be irrelevant in more elementary environments. Finally, given that participants are not interacting with real human opponents, we expect that the personality traits relevant for inter-human interaction (e.g., agreeableness, honesty–humility, extraversion) would not correlate with performance. Other personality traits contributing to cognitive tasks (e.g., conscientiousness, openness) may show an effect^22,23^.

The predictions on neural activity rely on the combination of our theory and the rich research on task processing in the brain. They provide an independent test of the theory we propose. We predict the involvement of the brain networks supporting the processing of the rules and facts relevant for deciding a course of action. These have been extensively studied in single-agent tasks (e.g., the Multiple Demand Network—MDN^24–33^). Regions within the MDN should be more active when participants deal with highly complex strategic environments, and when the relevant information needs to be updated. The individual cognitive ability should influence the response to critical task events in this brain network^34–37^. Moreover, the strategic nature of the games we use and the fact that the task is performed in a social context require the participants to be able to predict their partners’ choices to best respond to their predicted decision and maximize their own gain. The ability to consider others’ mental states and anticipate their actions is usually referred to as the Theory of Mind (ToM). This ability is associated with a brain network including regions like the medial prefrontal cortex, the precuneus, and the temporoparietal junction^38–40^. Typically, one would expect the involvement of this network during interactive decision-making. However, previous research has shown that the involvement of the ToM network during social tasks depends on the belief of being interacting with human partners as compared to artificial agents^41,42^. Since our participants’ opponents are not human, we do not expect to observe activity in this network.

## Methods

In the first part of the Methods section, we present the background necessary for the construction of the complexity and performance measures. We begin by introducing the task and the simulated opponents (or *automata*), we provide a measure of the complexity of strategies, and we derive the optimal policy of the subject and the associated loss function. Finally, we describe the experimental design and procedures and present the empirical strategy.

### The stochastic game and the stage games

In the experiment, subjects played several instances of a *stochastic game* against computerized opponents (or automata). A stochastic game is a sequence of matrix games, called *stage games*, played with the same opponent, which in our case followed the rules we are going to describe. The sequence of stage games has a random termination, as explained in points (2) and (4) below. The set of stage games *G* has two elements: a Prisoner’s Dilemma (*PD*) and a Battle of the Sexes (*BoS*) game, described in Tables 1 and 2 below. The transition among games in two successive trials is random and independent of the actions of the players. The stochastic game starts with an initial stage game belonging to the set *G*; at every new stage, if *g* is the stage game in the previous period, the stage game in the next period may be the same as *g*, or it may change to a new game $g' \ne g$ with some known probability. Before the play of any new stage game:

1. A choice is made whether the experiment continues (with probability $\delta \in (0, 1)$) or not.
2. Conditional on continuing, a choice is made of whether the type of opponent changes or not. The opponent changes with probability $1-\xi \in (0,1)$, so that $\xi$ is the probability of staying with the same opponent.
3. If the opponent does not change, a choice is made of whether the stage game changes or not; the game changes to the alternative one in the set with probability $(1-\rho ) \in (0,1)$, so that $\rho$ is the probability of playing the same stage game.
4. If the opponent changes, we can consider this the end of the current stochastic game, and a new one begins. A random choice of opponent is made over the set of possible automata, with a fixed uniform probability over the types. Each type starts at its fixed initial condition, as explained in the section Automata and States. Our experiment had six different opponents (distinguished by personal names) corresponding to three automaton types.

The random events described in the steps above, which we refer to as “choices of Nature”, thus determine the end of the experiment, the transition to a new opponent, and the transition to a new stage game.

The payoffs of the stage games (in Experimental Currency Units— ECU) are reported in Tables 1 and 2.

**Table 1:** Prisoner’s dilemma payoffs.

	$\hbox {Coop}_{2}$	$\hbox {Def}_{2}$
$\hbox {Coop}_{1}$	(48, 48)	(12, 50)
$\hbox {Def}_{1}$	(50, 12)	(25, 25)

**Table 2:** Battle of the sexes payoffs.

	$\hbox {Worst}_{2}$	$\hbox {Best}_{2}$
$\hbox {Best}_{1}$	(48, 25)	(0, 0)
$\hbox {Worst}_{1}$	(0, 0)	(25, 48)

Each cell indicates the outcomes for both players for a given pair of actions. In the *PD*, if both players cooperate (i.e., the pair of actions is $Coop_1, Coop_2$), each gains 48 ECUs; if they both defect ($Def_1, Def_2$), they gain 25 ECUs each; finally if one defects and the other cooperates, the former gains 50 ECUs and the latter 12 ECUs. In the *BoS*, the players obtain a zero payoff if they both choose their own best option $(Best_1, Best_2)$ or if they both choose their own worst option $(Worst_1, Worst_2)$. In the other cases, player *i* gains 48 ECUs if the players coordinate on *i*’s best option ($Best_i, Worst_j$) and 25 ECUs if they coordinate on *j*’s best option ($Worst_i, Best_j$), with $j \ne i$.

We use the generic notation $a \in D_1$ for the action of the human participant (player 1, i.e., the row player in the tables above) and $r \in D_2$ for that of the automaton (player 2, i.e., the column player in the tables above). The action set may be taken to be independent of the game, if we denote (in the case of player 1):

$$\begin{aligned} a&= C \, \text { for} \, Coop_1 \, \text {in} \, PD \, \text {and} \, Worst_1 \, \text {in} \, BoS \\ a&= A \, \text { for } \, Def_1 \, \text {in} \, PD \, \text {and} \, Best_1 \, \text {in} \, BoS \end{aligned}$$

with the following interpretation justifying the notation: *C* for cooperative behavior and *A* for aggressive behavior. We use the same labels for *r*.

### Automata and states

From the point of view of a human player, the current situation in the stochastic game can be minimally described as a *state* *s* in a set *S* that we now describe. Informally, the set *S* is the product of the external conditions set by the stochastic process, that is, the current stage game and opponent type, and the elements that determine the opponent choice, which we refer to as internal state of the automaton. We describe each component in detail.

Our automata have a finite number of internal states $\Omega$. The actions of the human participant and the choices of Nature determine the transition to a new state. Each automaton is characterized by two deterministic rules. First, a transition function $\tau : \Omega \times G \times D_1 \rightarrow \Omega$, that we call *state transition*. This function determines the new internal state $\omega ^{\prime } \in \Omega$ of the automaton, where $\omega ' = \tau (\omega , g, a)$, given the previous internal state $\omega$, the previous stage game *g* and the choice of the human player *a*. Second, a *rule of behavior*, a function $f:\Omega \times G\rightarrow D_2$, that determines the action $r\in D_2$ of the automaton, where $r = f(\omega ,g)$, given the current internal state $\omega$ and the current stage game *g*.

There are three possible types of simulated opponents (i.e., automata) in the experiment, namely *AD*, *GT*,  and *TfT* (see description below). The set of internal states for *AD* is a single element; the set $\Omega$ for *GT* and *TfT* has two states, *Retaliation* and *Not Retaliation*.

1. AD (Always Defect). *State Transition:* Irrelevant. *Rule of behavior:* Play *A*.
2. GT (Grim Trigger). *State Transition:* If in retaliation state (i.e., in the previous stage, the opponent played *A* in *PD* or broke coordination in *BoS*) in the last stage, stay in retaliation; if not in retaliation in the last stage, move to retaliation state if in the last stage (a) the stage game was *PD* and the other played *A* or (b) the stage game was *BoS*, coordination occurred in past stages, and coordination failed in the last stage. *Rule of behavior.* If in retaliation state, play *A*; if not in retaliation, play *C* in *PD*, and play *A* in the first stage of *BoS* and the action you did NOT play in the last stage in the following stages.
3. TfT (Tit for Tat). *State Transition:* Move to retaliation state and cancel the record of past coordination in *BoS* if (a) the stage game is *PD* and the other played *A* or (b) the stage game is *BoS*, coordination occurred in past stages and coordination failed in the last stage. *Rule of behavior.* If in retaliation state, play *A*; if not in retaliation, play *C* in *PD* and in the first stage of *BoS*, and in the following stages of *BoS* play the action you did NOT play in the last stage.

In other words, *AD* represents an aggressive opponent who never cooperates in the *PD* and always tries to reach their most preferred outcome in the *BoS*. *TfT* and *GT* represent opponents willing to cooperate in *PD*, and to alternate between their and the opponent’s preferred outcome in *BoS*. They differ in their retaliation strategies, which last only one round for *TfT* and last forever for *GT*.

We now assemble all the elements defining a state $s \in S$. We denote by $\theta \in \{AD, GT, TfT\}$ the type of the automaton, by $g \in \{ PD, BoS\}$ the stage game, by $x \in \{ 0,1\}$ a variable recording whether a coordination has taken place in *BoS*, by $y \in \{0, 1\}$ a variable recording whether the automaton is in retaliation state or not, and finally by $z \in \{ b, w\}$ a variable recording whether the automaton will play Best or Worst the next time, if the next game is *BoS*. A state is:

$$\begin{aligned} s\equiv & \, {} (\theta ,g,x,y,z)\\\in & \, {} \{AD, GT, TfT\} \times \{ PD, BoS\} \times \{0,1\} \times \{ 0,1\} \times \{b, w\} \equiv S \end{aligned}$$

This summary is the minimal information that is relevant for the decision-maker: it allows the participant to predict the action of the automaton in the current stage, the transition to the next state conditional on their own action, and so on. However, it is not necessary for every type of automaton, and, therefore, the state space can be considerably simplified, noting that:

1. the type *AD* has a single internal state and there is no need to record the *x*, *y* and *z* variables; just the current game suffices. Thus, only 2 states are necessary when $\theta =AD$;
2. the type *GT* changes into an *AD* automaton (and never comes back) when it enters the retaliation state; therefore, there is no need to keep track of the *y* variable, since the type keeps track of the retaliatory state; thus, only 8 states (plus the two for the *AD*/retaliation state) are necessary when $\theta = GT$;

In the notation indicating the states (only in point (1) and (2) below), we shorten *PD* and *BoS* to *P* and *B*, respectively, and *AD*, *GT*, *TfT* to *A*, *G* and *T*, respectively. We have:

1. 10 states having *AD* or *GT* as type of the automaton (*AD* is equivalent to *GT* in the retaliation state thus the two automata can be collapsed):
  $$\begin{aligned} \{Ag, Gg0b, Gg0w, Gg1b, Gg1w\} \end{aligned}$$
  for $g \in \{PD, BoS\}$; this subset of the simplified state space is invariant, until the type of the opponent changes.
2. 16 states having *TfT* as type of the automaton:
  $$\begin{aligned} \{ Tg00b, Tg00w, Tg01b, Tg01w, Tg10b, Tg10w, Tg11b, Tg11w \} \end{aligned}$$
  for $g \in \{PD, BoS\}$; this subset of the simplified state space is also invariant.

Finally, to define an automaton, we need to determine the *x* state in the first stage of the stochastic game (i.e., the initial conditions $x_0$ for that automaton). We use the following:

1. *ADg* when the type is *AD*, with *g* chosen randomly with equal probability;
2. *GTg*0*b* when the type is *GT*, with *g* chosen randomly with equal probability;
3. *TfTg*00*w* when the type is *TfT*, with *g* chosen randomly with equal probability.

Therefore, an automaton is a tuple $M=(\Omega ,f,\tau , x_0)$. Together with the rules governing the stochastic game, it fully describes the task administered to the participants in the experiment.

### Transition matrices

A transition probability map for each action *a* of the participant associates the probability of a future state $s^{\prime }$ to the current state *s*. We denote it by $T_{a}(s^{\prime }, s)$. The transition has two deterministic components: the rule of behavior of the automaton in the current state and the rule to update the internal state of the automaton given the state and the state transition (i.e., *a*). It also has one purely probabilistic component: the process that governs the continuation of the experiment and the evolution of the external state, that is, partner and stage-game change. From the counting in the section Automata and States (i.e., 10 states for *AD* and *GT* and 16 states for *TfT*), the resulting transition matrix representing $T_{a}(s^{\prime }, s)$ is a $26 \times 26$ matrix. Changing partners or finishing the experiment ends the stochastic game (the experiment ends with probability $(1-\delta )$; the opponent changes with probability $(1-\xi )$; both probabilities are independent of the participant’s action); therefore, we can disregard transitions to new partners. This gives the matrix a very simple structure: it can be decomposed in two sub-matrices, a $10 \times 10$ matrix for *AD* and *GT*, and a $16 \times 16$ matrix for *TfT*. This matrix is specific to the action of the participant. Since the participant has two actions, we have two of these matrices. The transition function can also be simplified by observing that also the game change is independent of the participant’s action. It follows that we can focus on the deterministic component given by the internal state, that is, the transition to the new combination $(x^{\prime }, y^{\prime }, z^{\prime })$ from the old one (*x*, *y*, *z*).

The tables that follow illustrate examples of these transition matrices. In each matrix, the row represents the old state, for the type of opponent relevant to that matrix, and the columns report the possible new state, assuming that the opponent does not change. Each cell represents the probability of a transition from the old to the new state, given the action of the participant. Since we are focusing on the deterministic component, these probabilities can only be zero or one. Below, we report an illustrative example when the action of the participant is *C* (so *Worst* in *BoS*) and the game is *BoS*. In the notation used in the transition matrices (and only there) we shorten *PD* and *BoS* to *P* and *B*, respectively, and *AD*, *GT*, and *TfT* to *A*, *G*, and *T*, respectively. In the example, the relevant transitions for each type of automaton are the following (the other transition matrices are straightforward modifications of the ones presented here and are reported in full in Supplementary Information  B):

- *AD*:
  $$\begin{aligned} \begin{array}{cc} &{} Ag^{'} \\ Ag &{} 1 \end{array} \end{aligned}$$
- *GT*:
  $$\begin{aligned} \begin{array}{cccccc} &{} Ag^{'} &{} Gg^{'}0b &{} Gg^{'}0w &{} Gg^{'}1b &{} Gg^{'}1w \\ Ag &{} 1 &{} 0 &{} 0 &{} 0 &{} 0 \\ GB0b &{} 0 &{} 0 &{} 0 &{} 0 &{} 1 \\ GB0w &{} 0 &{} 1 &{} 0 &{} 0 &{} 0 \\ GB1b &{} 0 &{} 0 &{} 0 &{} 0 &{} 1 \\ GB1w &{} 1 &{} 0 &{} 0 &{} 0 &{} 0 \end{array} \end{aligned}$$
- *TfT*:
  $$\begin{aligned} \begin{array}{ccccccccc} &{} Tg^{'}00b &{} Tg^{'}00w &{} Tg^{'}01b &{} Tg^{'}01w &{} Tg^{'}10b &{} Tg^{'}10w &{} Tg^{'}11b &{} Tg^{'}11w\\ TB00b &{} 0 &{} 0 &{} 0 &{} 0 &{} 0 &{} 1 &{} 0 &{} 0\\ TB00w &{} 1 &{} 0 &{} 0 &{} 0 &{} 0 &{} 0 &{} 0 &{} 0\\ TB01b &{} 0 &{} 0 &{} 0 &{} 0 &{} 0 &{} 1 &{} 0 &{} 0\\ TB01w &{} 0 &{} 0 &{} 0 &{} 0 &{} 0 &{} 1 &{} 0 &{} 0\\ TB10b &{} 0 &{} 0 &{} 0 &{} 0 &{} 0 &{} 1 &{} 0 &{} 0\\ TB10w &{} 0 &{} 0 &{} 1 &{} 0 &{} 0 &{} 0 &{} 0 &{} 0\\ TB11b &{} 0 &{} 0 &{} 0 &{} 0 &{} 0 &{} 1 &{} 0 &{} 0\\ TB11w &{} 0 &{} 0 &{} 0 &{} 0 &{} 0 &{} 1 &{} 0 &{} 0\\ \end{array} \end{aligned}$$

These matrices represent the deterministic component of the transition to a new state. To account for the probabilistic component, if the game does not change (i.e., $g^{\prime } = BoS$ in the example), it is sufficient to multiply them by $\delta \xi \rho$ (the probability that the experiment continues, that the opponent does not change, and that the game does not change, respectively). If the game changes (i.e., $g^{\prime } = PD$ in the example), it is sufficient to multiply them by $\delta \xi (1-\rho )$. If the opponent changes, which occurs with probability $1-\xi$, then the transition is to the state where the opponent is the new opponent, and the history is the initial history with that new opponent.

### Complexity measure

The complexity of the strategic environment that a human participant faces results from two components: the strategy of the opponent (i.e., the type of automaton that is currently playing) and the stage game. We examine each in turn and construct an overall complexity measure.

Complexity measures for automata are well known. The measure of complexity that we adopt here is based on the size of the transition matrices of the environment. The tables presented in the section Transition Matrices already illustrate the different complexity of the various automata. *AD* is the least complex: to represent the choices of the opponent, the player has to keep in mind that the automaton is in a single state, with the same action associated with the two games. The number of states for *TfT* is larger than that for *GT*, since for *TfT* one has to keep track of whether retaliation is in force or not. Thus, we can conclude that *TfT* is more complex than *GT*, which, in turn, is more complex than *AD*.

Similarly, looking at the stage game, *PD* is simpler than *BoS*. Implementing cooperation (when the opponent is *GT* or *TfT*) in *PD* requires playing a constant action, whereas to coordinate in *BoS* the player needs to remember that they have to switch between the two actions. By contrast, when the opponent is *AD*, *PD* and *BoS* have the same complexity because, in both cases, the opponent chooses one action irrespective of the choice of the participant.

From the previous analysis, we conclude that, for complexity,

$$\begin{aligned} BoS \succeq PD \end{aligned}$$

and

$$\begin{aligned} TfT \succeq GT \succeq AD. \end{aligned}$$

**Figure 1:**
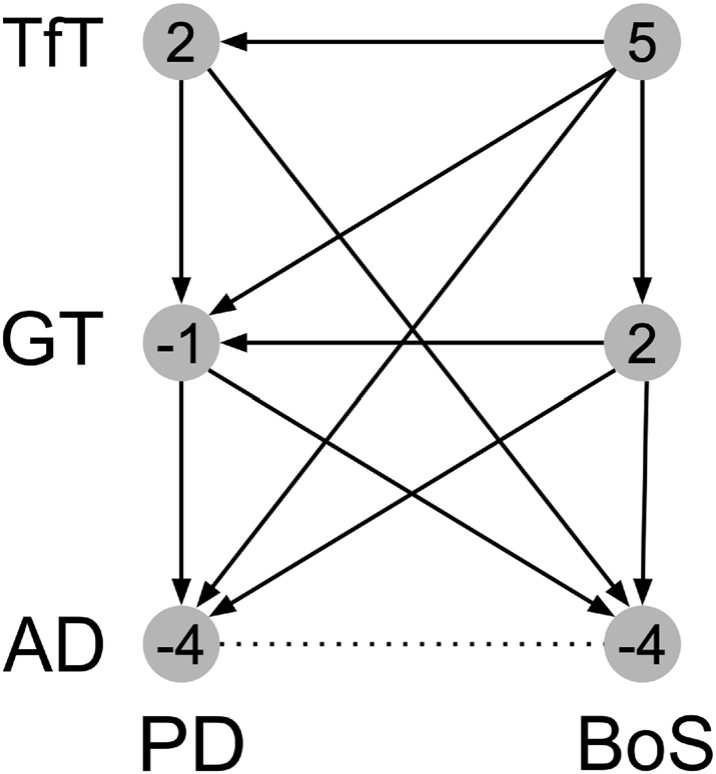
Predicted complexity relations. The vertices of the graph represent states, that is, pairs of opponent’s strategy and stage game. The directed edges represent the order, pointing to the least complex of the two vertices. The others follow taking the transitive closure. The number associated with each vertex is the measure of complexity associated with the corresponding pair of opponent and stage game, computed as the difference between the number of vertices it dominates and the number of vertices that dominate it.

We derive a partial order on pairs $(\theta , g)$ without further assumptions on possible interactions between the complexity in the two dimensions of game and opponent. In particular, we rank all pairs where one of the two components is the same or where one of the pairs is dominated by the other on both dimensions. For example, we can write $(BoS, GT) \succeq (PD, GT)$ or $(BoS, TfT) \succeq (PD, GT)$. Requiring the order to be transitive yields additional predictions that are presented in Fig. 1. Transitivity yields the edges going from top-right to bottom-left and the relation $(BoS, TfT) \succeq (PD, AD)$. As said above, $(AD,PD) \sim (AD, BoS)$. This, if we assume transitivity, yields the edges going from top-left to bottom-right. The resulting order among vertices in the figure is partial: for example, it does not tell us the order between (*PD*, *TfT*) and (*BoS*, *GT*).

Combining the complexity order among strategies (the vertical direction in Fig. 1) with that among stage games (the horizontal direction), we obtain an overall complexity measure. The combination we use assigns to a vertex *E* the number of vertices that *E* dominates, minus the number of vertices that dominate *E*. This net value is indicated by the numbers reported in Fig. 1, which is the complexity measure we use in the rest of the paper.

The complexity measure based on transition matrices is not the only one possible. As an illustrative example, it may be useful to consider a different measure, for example, the word length of the sentences describing the strategy. This proposed measure should satisfy the condition that the description of the automaton is minimal, in some sense to be made precise, or otherwise the measure might be altered by arbitrary, superfluous padding. If one agrees that the description provided is minimal, then the order induced by this measure is very similar to the one induced by the size of the transition matrix. However, providing a precise definition of a “minimal” description is hard, and this is one of the main reasons we chose the size of the transition matrix as a measure.

### Value function, optimal policy and loss

We first characterize the largest payoff a player can achieve, given an initial opponent and stage game. This will be used in our analyses as a benchmark to evaluate participants’ performance. We do not intend this model to be a descriptive model of the behavior of all the players; it may approximate the actual behavior of fully-rational, high-intelligence participants. It does, however, provide a benchmark that can be used to estimate the best payoff a subject can achieve. The main purpose of the model is to provide a theoretical prediction of the maximum payoff, and thus, once compared to the actual behavior, a measure of the loss incurred by the players. The main predictions of our theory are that the players incur higher losses the higher the complexity of the environment and that this relationship is stronger for players with lower intelligence. We assume participants know and understand the setup we have described and choose the best response within the constraints of their ability. The transition probability map and the reduced payoff function are the elements used to compute the value function. The problem is a standard dynamic programming problem, where the objective is to maximize the expected value of the game over the set of Markov policy functions $\pi : S \rightarrow D_1$:

1 $$\begin{aligned} V(s) = \max _{\pi } E \sum _{t=1}^{+\infty } (1-\delta ) \delta ^{t} u (s_{t}, a_{t}) \end{aligned}$$

under the condition that the initial state is *s*, and where *E* is the expected value operator. *V* is the unique fixed point of the Bellman operator:

2 $$\begin{aligned} B(\phi )(s) \equiv \max _{a \in A} \left( (1-\delta ) u(s, a) + \delta \sum _{s^{\prime }} T_{a}(s^{\prime }, s) \phi (s^{\prime }) \right) \end{aligned}$$

where $\phi$ is a function from *S* to the real numbers representing the candidate value function. Since $\delta < 1$, we can iterate the *B* defined in (2) to approximate numerically the true value function (so that $\phi = V$). Convergence is at an exponential rate, so we can estimate the number of iterations sufficient to ensure that the distance of the current iteration from the true value function is less than a prescribed value. In our computations, the probabilities of continuing with the same partner and of continuing with the same stage game are included in the transition probability map. Therefore, the discount factor only keeps track of the probability that the experiment continues. We set $\delta$ equal to 0.9 to match the average duration of the experimental session. The relative values are not substantially affected by the choice of different values, since changing them produces mostly a re-scaling of the values.

As we mentioned, the aim of the model is to produce an estimate of the loss associated with a behavior. We now describe how the variable *loss* is computed. First, we define a *Q* function as the sum of the current utility given an action and a state, and the continuation value from the state reached according to the action taken, the automaton, and the current state of the game; the sum is weighted by the discount factor:

3 $$\begin{aligned} Q(s,a) = (1-\delta ) u(s,a) + \delta \sum _{s^{\prime }} T_{a}(s^{\prime }, s) V(s^{\prime }) \end{aligned}$$

From the Bellman’s equation, for every *s* we have $V(s) = \max _{a \in A} Q(s,a)$, and we are interested in determining the loss at each pair (*a*, *s*)

4 $$\begin{aligned} L(s,a) = V(s) - Q(s,a). \end{aligned}$$

The loss is a non-negative real number, representing the cost of the error of choosing *a* at *s* (possibly zero, if the action chosen is optimal). For each participant, the *total loss* is the sum of the losses in each period: $\sum _{t: 1 \le t \le T} L(s_{t}, a_{t})$. By its definition, the total loss equals zero under the optimal policy. In the section The stochastic game and the stage games we described in detail the set of parameters. The experimental procedures, reported in the section Experimental stimuli and procedures, determine their values. As mentioned above, the probability of continuation, $\delta$, is set to 0.9; the probability of staying with the same opponent (conditional on continuation), $\xi$, is set to 0.75; the probability of playing the same game (conditional on staying with the same opponent), $\rho$, is set to 0.67. Finally, the running payoffs are those presented in Tables 1 and 2. With these parameter values, one can compute that the optimal policy is as follows:

- AD: Play *A* in *PD*; play *C* in *BoS*.
- GT: When in retaliation: Play *A* in *PD*; play *C* in *BoS*. When not in retaliation: Play *C* in *PD*; play *C* in the first stage of *BoS*; in the following stages of *BoS* (a) if you coordinated in the last stage, play the action you did NOT play in the last stage, (b) if you did not coordinate in the last stage, play the same action as in the last stage.
- TfT: Play *C* in *PD*. When not in retaliation: play *A* in the first stage of *BoS*; in the following stages of *BoS* (a) if you coordinated in the last stage, play the action you did NOT play in the last stage, (b) if you did not coordinate in the last stage, play the same action as in the last stage. When in retaliation: play *C* in *BoS*.

### Participants

Forty-two participants took part in the study in exchange for monetary payment. They were all German native speakers, right-handed (score $\ge 53$ at the Edinburgh Handedness Inventory^43^), with no reported psychiatric or neurological disorders nor uncorrected visual impairments, except one participant who was not a native German speaker and was ambidextrous. All experimental materials were in German. The sample consisted of 23 women and 19 men, with a mean age of 27.3 years (range 18–35). The experiment was approved by the ethics committee of the Humboldt University of Berlin and conducted in accordance with the Declaration of Helsinki. Participants signed the informed consent form before enrolling in the study.

### Experimental stimuli and procedure

**Figure 2:**
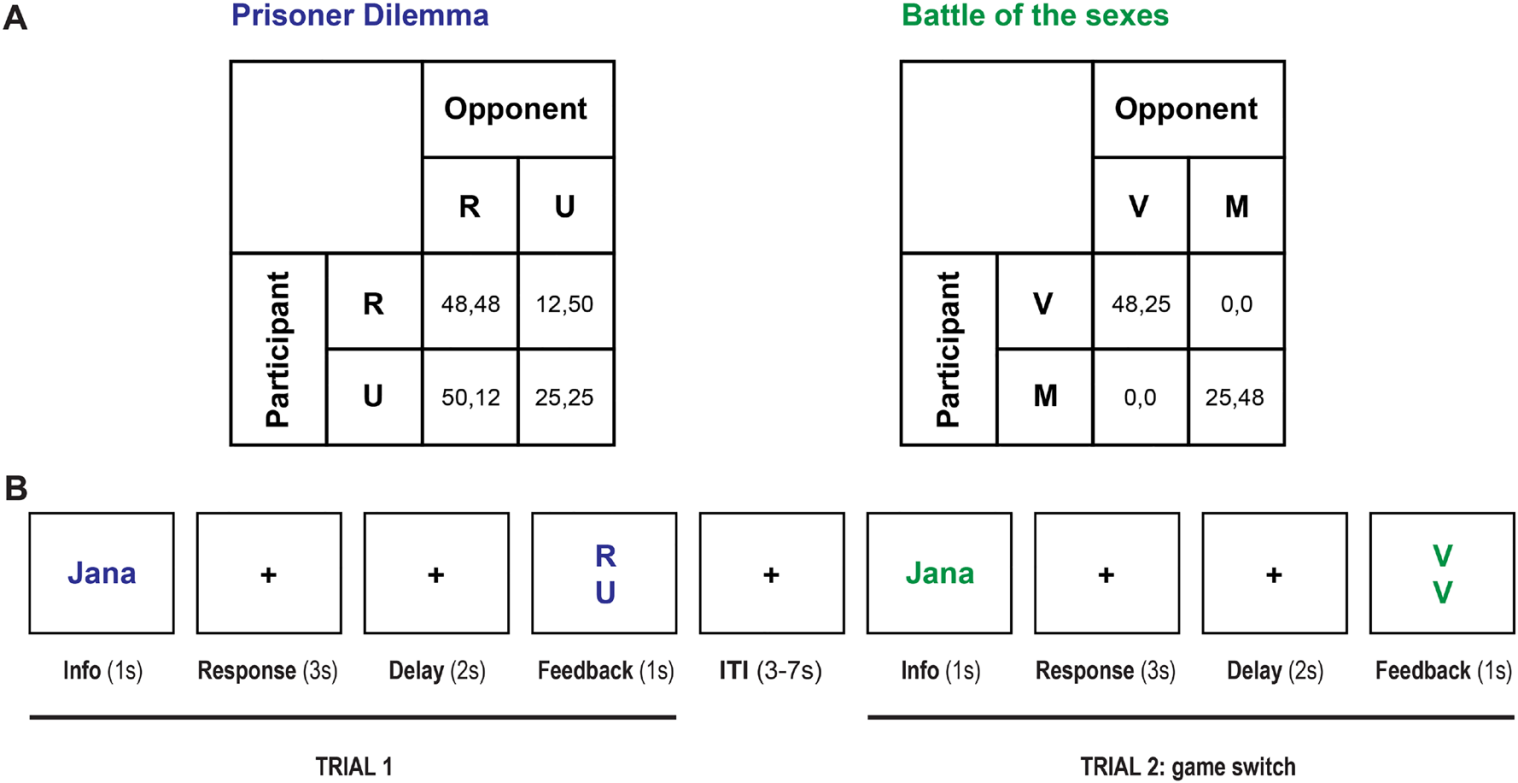
Experimental paradigm. (**A**) Payoff matrices for the two stage games (i.e., *PD*, *BoS*); payoffs are displayed in experimental currency units, for each game. The response options (i.e., R/U and V/M) displayed in the table correspond to those shown in Tables 1 and 2 (i.e., *Coop*/*Def* and *Best*/*Worst*, respectively). During the experiment, letter pairs were used. The stage game was indicated by the color (i.e., blue/red for *PD* and green/yellow for *BoS*, counterbalanced across participants). (**B**) An example of two consecutive trials in one *f*MRI session. In this example, the player (i.e., Jana) does not change between trials, but the stage game does (from *PD* to *BoS*), as shown by the change of color. During the first trial, the participant chose R (i.e., *Coop*) and the automaton U (i.e., *Def*), while during the second trial both the participant and the automaton chose V (i.e., the participant’s favorite option). *ITI* inter-trial interval.

Participants played the stochastic game described in the previous subsections while undergoing functional magnetic resonance imaging (*f*MRI) against one of six virtual players. A personal name identified each virtual player and was associated with a single and stable automaton, *AD*, *TfT*, or *GT*. The implementation of the strategy by the automata in the experimental design has no error. Each automaton was assigned to exactly two virtual players. Figure 2, panel A shows the payoff matrices shown to the subjects (in Experimental Currency Units—ECU) and an illustrative example of two trials. During the experiment, we showed the response options to the participants using different labels than those shown in Tables 1 and 2 to preserve their neutrality and help them distinguish between the two-stage games.

We generated a pseudo-random sequence of game and partner changes for each scanning run. We constrained the sequence so that the continuation probabilities for the partner and the game were both 50%. Partner and game change never occurred in the same trial. Overall, if you played 100 trials in about 50 you would play the same game with the same partner, in about 25 you would change the game, and in about 25 you would change the partner. Participants were given a detailed description of this procedure in the initial training session and briefly reminded of it at the beginning of the scanning session.

At the beginning of each trial (Fig. 2, panel B), a name printed in color appeared on the screen for 1 s. The name defined the virtual partner playing in the current trial (e.g., Jana), and the color indicated the current game (e.g., *PD*). The participants had to report their choices by pressing one of two buttons within 3 s. A blank screen was presented for 2 s after the choice was made. When the game was *PD*, the participants could use their index fingers to press the inner button of the left button box to choose to cooperate or the inner button of the right button box to choose to defect. In *BoS* they used the middle finger to press the external button on the left button box to choose their favorite option or the external button on the right button box to choose their non-favorite option. Finally, feedback about the other player’s choices was provided for 1 s. A variable inter-trial interval (ITI, range 3–7 s, mean 5 s) preceded the beginning of the next trial. During each scanning run, participants played 48 trials; in 24, both the game and the player remained the same as in the previous trial; in 12 trials, only the game changed; in the remaining 12 trials, only the player changed. To determine the participant’s final payment, the ECUs gained on each run were summed and converted into euros (300 ECUs = 1 euro) at the end of the experiment.

Before the *f*MRI session, participants underwent a training session scheduled at most four days before. At the beginning of the training, participants performed the 20-min timed version of the Raven’s Advanced Progressive Matrices^44^ (details in Section  A.1 of Supplementary Information), filled in a personality questionnaire^45^, and the ten items of the Honesty–Humility scale of the HEXACO-PI-R^46^. We chose a wide set of personality tests because there is still no consensus on the relationship between individual traits of subjects and strategic behavior, not even on which traits affect such behavior^47,48^. In an environment similar to the one used here, Proto and colleagues^5^ identified a weak and transitory role of Agreeableness and Conscientiousness, in addition to the role of intelligence. After completing the intelligence and personality tests, on which participants received no feedback, the participants familiarized themselves with the payoff matrices of the two games (*PD* and *BoS*) and the structure of the stochastic game. They were informed that they would be playing with different virtual players whose choice strategies were derived from real players’ choices. Then, they practiced the experimental task to familiarize themselves with the games and the virtual players. Each virtual player kept the same name and the associated automaton in the training and the *f*MRI session. The training session lasted, on average, 105.9 min (*SD* = 15.6 min). We admitted to the *f*MRI session only participants who demonstrated to have learned the virtual players’ strategies (i.e., met at least 3 out of 6 performance criteria related to the percentage of defection choices in *PD* and synchronized choices in *BoS* for the different strategies, assessed over the last 200 trials of the training). Overall, six participants were excluded because of poor performance during the training.

### Behavioral analysis

Our behavioral analysis tested whether the complexity of the strategic environment affected performance and whether the performance pattern changed systematically across individuals with different cognitive abilities or personality. We derived three main expectations from our model. First, the performance should be inversely correlated with the complexity of the environment, which is mainly determined by the game and the opponent’s strategy. We should also observe a drop in performance when the strategic environment changes and a reassessment of the situation is required. The second prediction relies on the idea that participants with a higher cognitive ability should deal more effectively with the most complex strategic environments. Therefore, while all participants may be affected by complexity, we expect that those with lower cognitive ability would be more affected than those with higher cognitive ability (i.e., they would show a disproportionate drop in performance in the most complex environments). Third, given the absence of interaction with human opponents, we expected that personality traits unrelated to cognitive skills would not correlate with behavior.

To test these predictions, we planned to perform a set of linear mixed-effect model analyses with random individual effects. The dependent variable was either the loss or the reaction time (RT) in a given trial. The target explanatory variable was the complexity index associated with each game combination (*PD*, *BoS*) and strategy (*AD*, *GT*, *TfT*). We ran the regressions on the whole sample and separately for change (strategy or game change) and no-change trials. We then focused on between-subject variability. The relation between intelligence and some of our dependent variables (loss, RT, activation) cannot be safely assumed to be linear. The relation could be, for example, U-shaped. The most intelligent participants may be fast and accurate, the average participants may be slower and accurate, and the least intelligent ones may not be accurate but fast because they use shallower strategies (see Figure A2 in the Supplementary Information, which provides evidence along these lines). Assuming linearity between Raven scores and the dependent variables would not be able to capture this (and other) nonlinear patterns. Thus, given the uncertainty about the shape of the relationship between cognitive ability and some dependent variables, we preferred to formulate a more open analysis approach based on a research question such as: “Is there any difference in performance/activation between participants with different levels of cognitive ability?”. We thus split the participants into three subgroups, using as cutoffs the 33 and 66 percentiles of the Raven score distribution. In this way, we generated a grouping variable based on the Raven score (the subgroups were uneven due to the distribution of the scores: low, n = 16; medium, n = 14; high, n = 12). The distribution of the Raven scores and the relevant cutoffs are reported in Fig. A1 (Supplementary Information). We added an interaction between this grouping variable and the complexity index to test for specific between-subject effects. We applied a statistical threshold of $p<0.05$, with standard errors clustered at the individual level to address the non-independence of observations for the same subject. The code used to perform the analyses is available at https://osf.io/h4n5c/.

### Neuroimaging analysis

The general aim of the *f*MRI analyses was to evaluate where and how the brain responds to variations in the complexity of the environment and, most importantly, to assess whether modulations in brain activity followed the pattern predicted by our model (i.e., reflected the complexity index), thus providing support for the theory we propose here. Furthermore, we investigated whether these modifications differed depending on the participants’ cognitive ability.

#### Image acquisition and pre-processing

*f*MRI data were recorded on a 3 T Siemens Trio scanner (Erlangen, Germany) equipped with a 12-channel head coil. We acquired T2$^{*}$-weighted images using a gradient-echo echo-planar imaging sequence. Six runs with 278 volumes (33 slices, 3 mm thick, with a gap of 0.75 mm, in-plane voxel resolution 3 mm$^{3}$) were recorded for each participant. The imaging parameters were: TR 2 s, TE 30 ms, FA $78^{\circ }$, matrix size 64 $\times$ 64, and FOV of 192 mm. We also acquired a T1-weighted anatomical scan and field mapping images. Imaging parameters for the anatomical dataset were: TR 1.9 s, TE 2.52 ms, FA 9, matrix size 256 $\times$ 256 $\times$ 192, FOV 256 mm $\times$ 256 mm $\times$ 192 mm, 192 slices (1 mm thick), resolution 1 mm $\times$ 1 mm $\times$ 1 mm. Acquisition parameters for the field mapping images were: TR 0.4 s, TE 5.19 ms and 7.65 ms, FA $60^{\circ }$, matrix size 64 $\times$ 64, FOV of 192 mm $\times$ 192 mm, 33 slices (3 mm thick), resolution 3 mm $\times$ 3 mm. *f*MRI data were pre-processed and analyzed using SPM12 (Wellcome Trust Centre for Neuroimaging, Institute of Neurology, University College London, London, UK). The functional images were realigned and slice-time corrected, geometric distortions were corrected using unwrapped field maps, and low-frequency noise was removed with a high-pass filter (cutoff period 128 s). Data were adjusted by fitting an autoregressive model to the residuals to allow for temporal auto-correlations. Functional images were co-registered to the anatomical scan, which was segmented using the template tissue probability maps in SPM12. Finally, the functional images were normalized to standard Montreal Neurological Institute (MNI) space and spatially smoothed with a 6 mm FWHM Gaussian kernel.

#### Whole-brain analysis

We implemented a whole-brain Finite Impulse Response (FIR) model for our first-level analysis. We set the temporal parameters of the FIR model to cover the duration of a single trial, from the presentation of the cue to the post-feedback elaboration. The moment of cue presentation was used as the onset time for all events. Each FIR time bin lasted one TR (i.e., 2 s), and each event was modeled with nine time-bins, thus covering 18 s from the onset time. The main FIR model included 12 conditions: 2 stage games (*PD*, *BoS*) $\times$ 2 trial types (change of the strategic environment, no change) $\times$ 3 partner strategies (*TfT*, *GT*, *AD*). We used FIR modeling instead of standard Hemodynamic Response Function (HRF) modeling to better follow the temporal unfolding of the neural response during a trial. FIR modeling also allowed us to better separate the neural responses associated with the two critical events in a task: the cue and feedback delivery. Considering the delay of the hemodynamic response, we assumed that cue-related neural activity would start at the second time bin (i.e., after 2 s from cue delivery), and it would reach the peak at the third or fourth time bin. Analogously, we associated with time bins from 5 to 7 the neural activity related to feedback delivery (see Section A.3 and Fig. A4 in Supplementary Information for a check of these assumptions). Notwithstanding the application of FIR modeling, separating the neural activity associated with cue and feedback delivery has limitations in the current task design. Given that (1) the conditions assigned to cue and feedback of a trial are necessarily always the same, and (2) the canonical hemodynamic response function lasts longer than 6 seconds, we cannot exclude that some activations, which are cue-related, would extend to the time window assigned to the feedback phase. On the other hand, we can at least affirm that: (1) activation observed during the cue phase cannot belong to the feedback phase (which temporally follows the cue phase); (2) activations observed during the feedback phase that are different or higher than those in the cue phase are most likely attributable to feedback processing. In the Supplementary Information, we report an accessory FIR analysis checking our assumptions on the coupling between neural activations and task events. In this analysis, we set up a model considering only right and left button presses. Given the relatively fast RTs we observed in the task, evidence of motor activation is a reasonable proxy to detect the beginning of the cue phase. Our assumptions were confirmed: The earliest signal of motor activity is detectable in the second time bin and peaks at the third time bin (see Section A.3 in Supplementary Information for details). To answer our main research question, we tested whether the observed neural activity across all conditions was in line with the theoretical predictions. For this purpose, we computed the measure of complexity (calculated using the theoretical model as explained in the section Complexity measure) associated with each task condition and assessed its correlation with the level of neural activation estimated with the first-level analysis for each task condition. Such correlation was computed across subjects for each time bin and both for change and no-change trials. We asked where in the brain the correlation was significantly higher than 0. We considered linear combinations of time bins related to the cue (time bins 2–4) and feedback (time bins 5–7) delivery. Additionally, we tested whether correlations were different in the two trial phases. Besides, as additional analysis, we explored which brain regions are involved in our task by using an alternative approach, relying on a different set of assumptions. This analysis assumed that the critical cognitive processes under investigation are more taxed upon strategic environment change, a common supposition in the task switching literature^49,50^. For example, when the environment changes, the participant needs to appraise the new situation, possibly recollects relevant information, generates a new responding strategy, determines the response, and evaluates the response at feedback. These processes are more (or only) involved when the strategic environment changes, with the likely exception of strategy application required for all trials. If this assumption is correct, we should observe the activation of a network similar to the one found by the primary analysis. We asked where in the brain change trials produced higher brain activations than no-change trials, collapsing across all other conditions (game and partner’s strategy). Finally, we tested for potential differences in the neural response between groups with different levels of cognitive ability. As for the behavioral analyses, we split our participants’ sample into three subgroups showing low, medium, and high cognitive ability based on two alternative measures (i.e., total loss and Raven test score). We tested whether the neural response to the task differed across the three subgroups in two independent analyses. More specifically, we tested for differences between subgroups in how well the actual pattern of neural activity (across conditions) reflected the theoretical predictions. The analyses were implemented as an interaction between group membership and the correlation computed in the primary neuroimaging analysis (see above). Whole-brain statistical inferences were based on a random-effects approach^51^. For all group-level analyses, we applied a statistical threshold of $p < 0.001$ at the voxel-level, uncorrected for multiple comparisons, and of $p < 0.05$ at the cluster level, family-wise error corrected for multiple comparisons^52^, following recommendations for proper cluster-level family-wise error correction in *f*MRI analysis^53^. All scripts used to perform the neuroimaging analyses are available at https://osf.io/h4n5c/.

#### ROI analyses on a-priori networks

**Figure 3:**
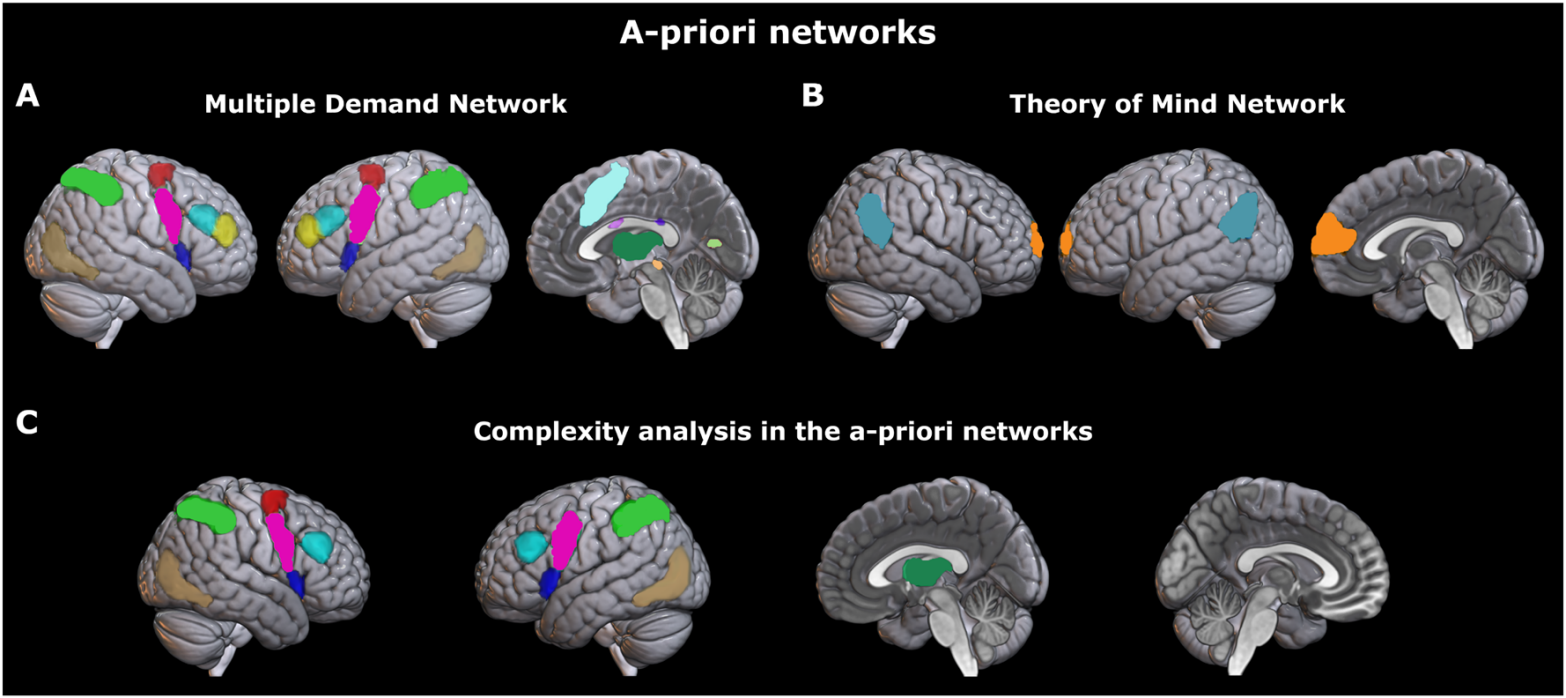
Effects within the multiple demand and the theory of mind networks. The effect of the environment complexity was tested in brain areas belonging either to the MDN (**A**) or the ToM network (**B**). While an effect was present in most of the MDN ROIs (**C**), it was absent in all the ToM ROIs. In the brain maps, colors are simply illustrative and define distinct ROIs in the MDN (**A**) and ToM network (**B**). For clarity, we depicted corresponding areas in the left and right hemispheres using the same color (**A**,**B**). (**C**) displays the ROIs from (**A**,**B**) in which the effect was significant (i.e., only the ROIs showing a significant effect). The color of the ROIs in (**C**) matches the color in (**A**,**B**).

The ROI analysis grants two important advantages: first, it allows for testing a-priori anatomo-functional hypotheses, and second, it increases the statistical power of the a-priori analysis. On the other hand, the following ROI analyses are not aimed at showing that the effect under study is anatomically specific, that is, mostly confined to the target network. For the latter aim, whole-brain analyses are more appropriate. Thus, the two analysis approaches are complementary and both informative. We tested the involvement of two a-priori networks potentially relevant for our task (see Introduction): the Multiple Demand Network (MDN) and the Theory of Mind (ToM) network. For the MDN^54^, we used the Region of Interest (ROI) maps (see Fig. 3, panel A) available on the MRC-CBU web site (http://imaging.mrc-cbu.cam.ac.uk/imaging/MDsystem). We only considered the ROIs larger than 50 voxels, and we excluded the ROIs in the cerebellum. To define the ToM ROIs, we relied on a systematic meta-analysis of *f*MRI studies^38^. We considered four ROIs in the Brainnetome Atlas^55^ (see Fig. 3, panel B) corresponding to the four regions displayed in Fig. 3C^38^: rostroventral area 39 (A39rv) and medial area 10 (A10m), bilaterally. We reran the whole-brain analyses described above using an a-priori ROI approach. For each ROI and each participant, we extracted the mean correlation between theoretical parameters (i.e., the complexity index) and observed activations. As in the whole-brain analysis, we tested whether correlations were significantly higher than 0 or differed across subgroups with different cognitive abilities. We corrected for multiple comparisons using the Bonferroni correction in all ROI analyses so that the overall $\alpha$ level remained below 0.05. Finally, to assess the differential effect of the network (MDN vs. ToM) on brain responses in different complexity conditions, we ran a linear mixed-effects model (LMM) analysis. We implemented a model with neural activation as predicted value and network and complexity as fixed effects. We introduced intercepts for subjects as random effects to account for individual variability in mean neural response. The LMM analysis was performed using R 4.1.0 (The R Foundation for Statistical Computing, Vienna, Austria) and the lme4 package^56^ for LMM analyses^57^. The *p* values of the statistical tests were calculated by performing likelihood ratio tests contrasting the full model including the effect of interest and the model without this effect^58^.

## Results

### Behavioral results

Behavioral analyses provide the first test of our theory.

#### Environment change and environment complexity

A first descriptive analysis of the data showed that subjects chose according to the optimal policy 84% of the time. In *PD*, they achieved mutual cooperation with *TfT* and *GT* in 77% of the rounds and mutual defection with *AD* in 92% of the rounds. In *BoS*, they coordinated on *AD*’s preferred outcome 94% of the time. They managed to coordinate in alternating between the two options at a rate of 72% with *TfT* and of 51% with *GT*.

We predicted that the strategic complexity would affect performance measures: subjects should show higher loss and longer RTs when complexity is greater. Furthermore, during the task, the strategic environment changed and the subjects had to adapt to such variations: whenever the game or the opponent’s strategy changed, a reassessment of the current situation was necessary. Subjects had to determine, or retrieve from memory, how to deal with the new situation. Such adaptations required additional cognitive processing that should produce longer RTs and higher loss upon environment change. To test these predictions on behavioral measures, we performed a set of mixed-effect model analyses with either the loss or the RT in a given trial as the dependent variable, the index of complexity as the main fixed effect, and individuals as the random effect.

Complexity had a positive and significant effect on both loss ($\chi ^2(1)=74.02; p<0.001$) and RTs ($\chi ^2(1)=60.40; p<0.001$; see Columns (1) and (2) of Table A1 in Supplementary Information). In terms of magnitude, the estimates on the whole sample imply that the loss doubles when moving from the simplest conditions where the opponent’s strategy is *AD* to the next more complex condition, where the opponent is *GT* and the game is *PD*. It is predicted to double a second time when moving from there to the most complex condition, where the opponent is *TfT* and the game is *BoS*. For the RTs, the estimates predicted an increase of about 150 ms along the range of complexity experienced in the experiment. Regressions on switch and stay trials (Columns (3)–(6)) showed that these effects were larger when either the opponent or the game changed than when the environment stayed the same as in the previous trial.

#### Inter-individual variability

We expected that environmental complexity impacted differently on people depending on their cognitive ability. Namely, we expected a disproportionate increase of loss in the most complex environments (e.g., *TfT*/*GT* in *BoS*) in subjects with a lower cognitive ability. We checked these expectations by splitting the subjects into subgroups. To explore the effect of between-subject variability on performance, we generated three subgroups based on the Raven score. We ran two further mixed-effect model analyses with either the loss or the RT as the dependent variable. We included an interaction between the index of complexity and the subgroup to which the individual belonged. In this way, we could test whether an increase in complexity affected more the performance and the RTs of subjects in the low, medium, or high Raven-score group. Full results are shown in columns (7) and (8) of Table A1 in Supplementary Information.

**Figure 4:**
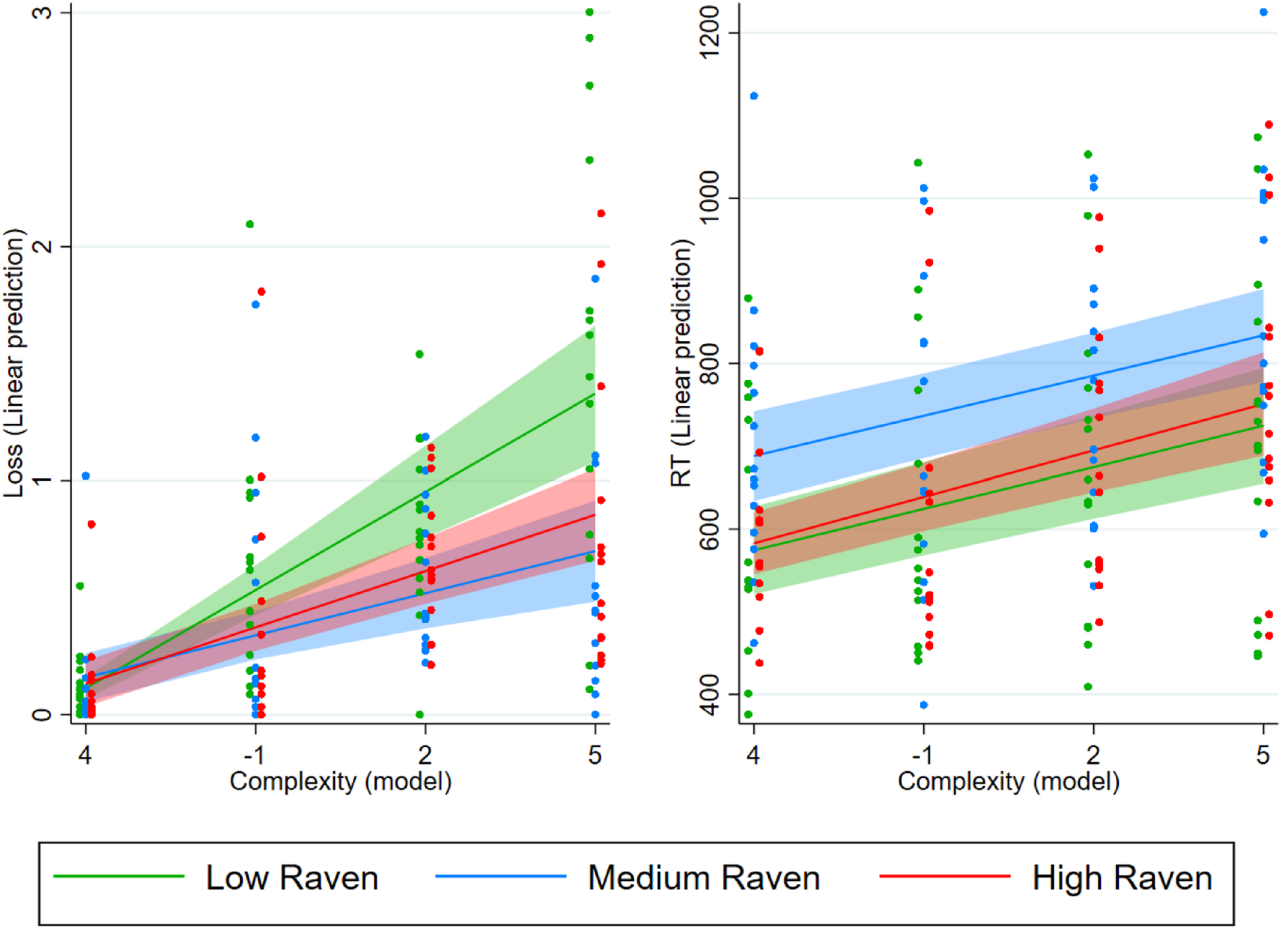
Complexity, Raven and behavior. Marginal effects of complexity on loss and RT from mixed-effect model analysis for groups with low, medium, and high Raven scores (individual data points overimposed).

Complexity increased loss for all groups (low: $\chi ^2(1)=51.99; p<0.001$; medium: $\chi ^2(1)=14.36; p<0.001$; high: $\chi ^2(1)=33.85; p<0.001$). However, it affected the low Raven-score group significantly more than both the medium ($\chi ^2(1)=10.12; p<0.01$) and the high Raven-score groups ($\chi ^2(1)=6.22; p=0.013$). The left-hand panel of Fig. 4 shows the predicted loss of the three groups as a function of the level of complexity. In the least complex trials, the three groups had a similar performance. Due to the differential effect of complexity between the three groups, in the most complex conditions the predicted loss was almost twice as large for the low Raven-score group as it was for the other two groups. Complexity also increased RTs for all groups (low: $\chi ^2(1)=24.99; p<0.001$; medium: $\chi ^2(1)=20.46; p<0.001$; high: $\chi ^2(1)=19.93; p<0.001$). It did so in a similar way across all Raven-score groups (low vs. medium: $\chi ^2(1)=0.01; p=0.919$; low vs. high: $\chi ^2(1)=0.14; p=707$; medium vs. high: $\chi ^2(1)=0.21; p=0.649$). The right-hand panel of Fig. 4 shows the predicted RTs of the three groups as a function of the level of complexity. While an increase in complexity was associated with a similar increase in RTs across the three groups, the level of RTs was not the same across groups. The medium Raven-score group showed higher RTs than both the low ($\chi ^2(1)=3.45; p=0.058$) and the high Raven-score groups ($\chi ^2(1)=4.49; p=0.032$) for all degrees of complexity. Thus, the medium Raven-score group had a similar performance as the high Raven-score group in terms of loss. However, this performance was achieved through longer RTs for the medium Raven-score group. RTs in the low Raven-score group, instead, were similar to those of the high Raven-score group, but the performance of the former deteriorated in relative terms as complexity increased. We obtain qualitatively similar results when studying the interaction between complexity and (continuous) Raven scores when we allow for a non-linear relation between intelligence and the dependent variables (see Figure A3 in the Supplementary Information).

Besides cognitive ability, we also tested whether personality factors were associated with modifications in the pattern of choices observed in our task. We collected six main personality factors: the Big Five (Openness, Conscientiousness, Extraversion, Agreeableness, Neuroticism)^59^ and Honesty-Humility^46^. For each factor, we adopted the same approach we used for the Raven analysis: we divided the whole group of subjects into three subgroups with cut-offs based on the 33 and 66 percentiles. We then assessed the presence of an interaction between any personality factors and our measure of complexity. All 36 interactions were non-significant at the alpha level of 0.05, even when correction for multiple comparisons was not applied.

### Neuroimaging results

#### Overview of the neuroimaging analysis

The neuroimaging analyses had multiple aims. First and foremost, we assessed whether the neural response to strategic complexity followed the pattern derived from our model. Second, we tested whether the response pattern to strategic complexity differed depending on the participants’ cognitive ability. Third, we sought to replicate the results of our primary analyses using an approach relying on an alternative set of methodological assumptions. Fourth, we investigated whether our main findings could be replicated in two a-priori brain networks potentially relevant for our task: the Multiple Demand Network and the Theory of Mind network.

#### Consistency with predictions derived from our model

**Figure 5:**
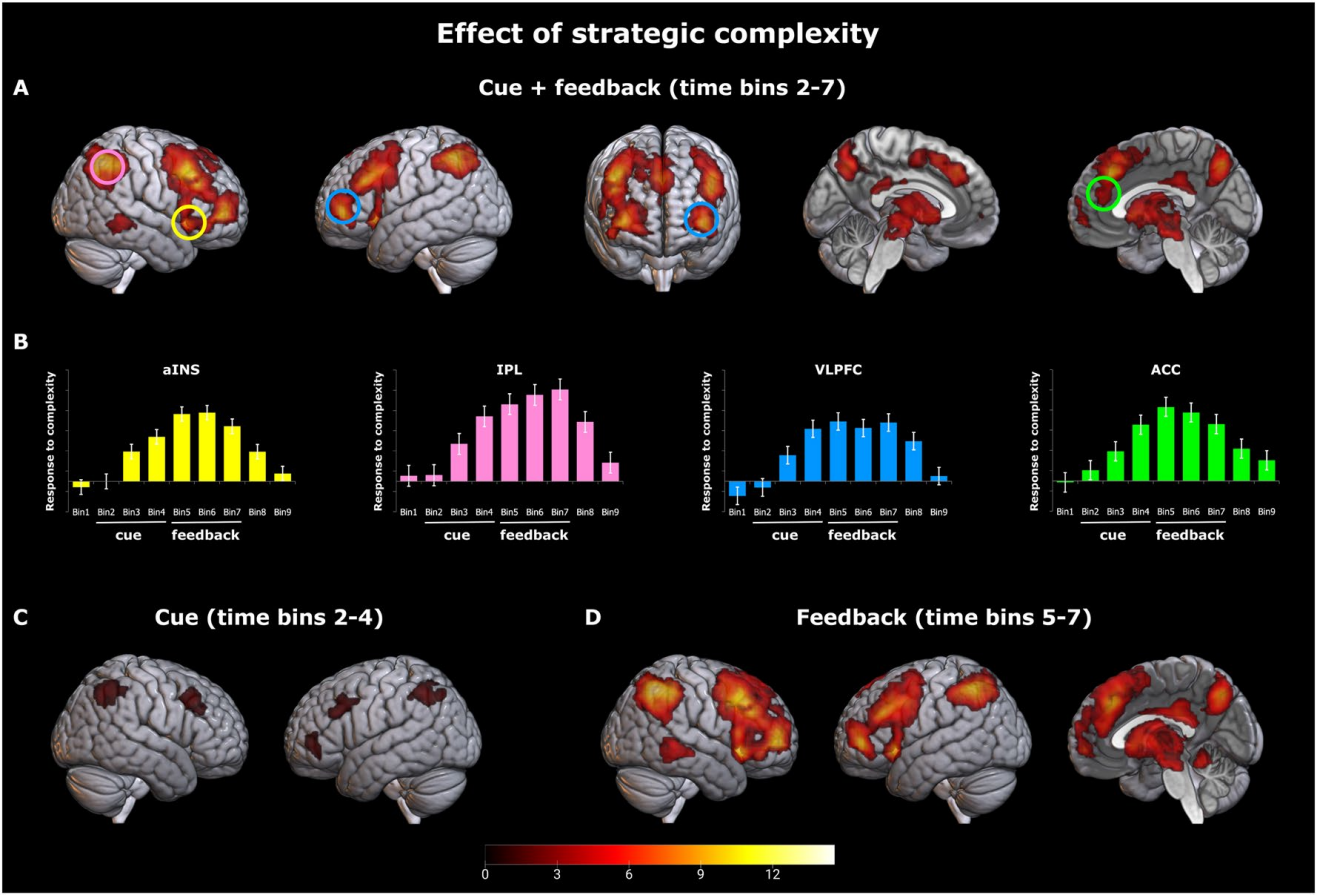
Effect of strategic complexity. Brain areas where neural activation correlated with strategic complexity during both cue and feedback phases (**A**), or only upon cue (**C**), or feedback (**D**) presentation. Modulation of the neural response produced by variation in strategic complexity (**B**). The relation between neural response and complexity is displayed over time in four example peaks in the anterior insula (first graph from left), inferior parietal lobule (second graph), ventrolateral prefrontal cortex (third graph), and anterior cingulate cortex (fourth graph). The x-axis of the bar-plots shows the time bins, each lasting 2 s. The time bins likely corresponding to the cue and feedback phases are highlighted. The y-axis shows the intensity of the modulation of the neural response produced by the strategic complexity, that is, the average coefficient of regression of complexity. In (**A**), the position of the example peaks is circled (with a color matching the corresponding graph). Error bars represent SEM. Intensities in (**A**,**C**,**D**) are color-coded according to the color bar displayed at the bottom, representing the correspondence between *t*-values and colors. We used a threshold of 0.001 at the voxel level and of 0.05 at the cluster level, FWE-corrected for multiple comparisons. *aINS:* anterior insula, *IPL:* inferior parietal lobule, *VLPFC:* ventrolateral prefrontal cortex, *ACC:* anterior cingulate cortex.

We tested whether the relationship between brain activation and strategic complexity followed the expectations derived from our model, that is, whether brain activity reflected the complexity index described in Methods. We observed a robust correlation ($p<0.05$, corrected) in a broad network involving predominantly medial and lateral fronto-parietal regions (Fig. 5, panels A and B, see Supplementary Information for the statistical tables).

We then explored whether the relation between complexity and neural activation was modulated by trial type (i.e., change vs. no-change trials), for example, by displaying a stronger correlation in change trials. The pattern did not differ significantly across trial types: the brain network involved in the two conditions was very similar. We explored whether the relation between complexity and neural activation was modulated by task phase. We observed a difference between the two task phases, cue and feedback. At the cue phase (i.e., time bins from 2 to 4), a relatively confined set of fronto-parietal regions showed a pattern of activity consistent with the theoretical expectations (Fig. 5, panel C). At the feedback phase (i.e., time bins 5 to 7), a much wider network emerged, overall similar to the network observed in the main analysis (Fig. 5, panel D). We further tested whether these apparent differences in the simple contrasts of the two task phases were statistically significant even when the two phases are directly compared ($p<0.05$, corrected). The majority of the brain regions showed a stronger correlation during feedback than cue phase. These observations suggest that the observed regions react more (or only) to strategic complexity in the feedback phase.

#### Inter-individual variability

**Figure 6:**
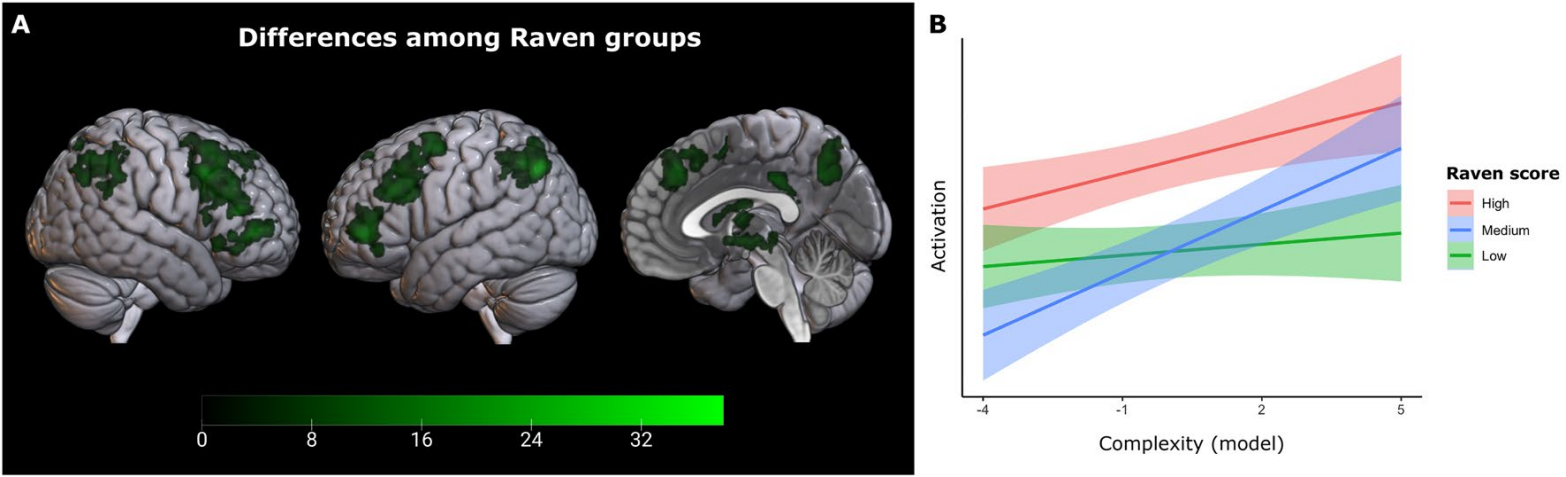
Brain response to strategic complexity in different Raven subgroups. Brain regions showing a different relation with strategic complexity in the three Raven subgroups (**A**). Relation between complexity and activation in the three Raven subgroups with high, medium, or low Raven scores (**B**). In (**B**), we averaged the signal in all regions reported in (**A**), that is, those regions showing a subgroup effect. The areas in (**A**) are color-coded according to the color scale below the maps showing the corresponding *F*-values. Areas on the map are thresholded at 0.001 at the voxel level and at 0.05 at the cluster level, FWE-corrected for multiple comparisons.

Similarly to the behavioral analyses, we expected that the complexity of the environment affects brain activation differently depending on the participants' level of cognitive ability. To assess this expectation, we tested whether the observed correlation between activation and strategic complexity would differ in people with higher, medium, or lower cognitive ability, as measured by the Raven test. We focused our analysis on the brain regions detected in our primary analysis on strategic complexity, where the whole sample of participants was considered. Thus, we applied a Small Volume Correction to limit our search volume to the regions displayed in Fig. 5. The correlation between strategic complexity and activation differed among subgroups ($p<0.05$, corrected). What explains the interaction between complexity effect and Raven group? We examined the brain activations across complexity levels for each subgroup of participants. We extracted and averaged activations from the whole brain network showing a subgroup effect (Fig. 6, panel A). The complexity of the environment barely modulated the neural activations in the subgroup with the lowest Raven score, while both the other groups showed a linear increment in activation as complexity increased (Fig. 6, panel B).

#### Change of the strategic environment

To check the robustness of our findings, we additionally employed an alternative approach to uncover brain regions involved in our task. This additional analysis relied on a different set of methodological assumptions, thus granting a further check on the results of the primary analysis. In this case, we assumed that the critical cognitive processes under investigation are more taxed upon change of the strategic environment. If this is correct, we should observe the activation of a network similar to the one we found with our primary analysis when comparing the activation between change and no-change trials, averaging across complexity levels. This analysis is statistically independent from the primary analysis on complexity effects. The results of this supplementary analysis confirmed those of the primary analysis (see Fig. A5 in Supplementary Information): areas where brain activation was higher in change than no-change trials form a fronto-parietal network very similar to the one reported in Fig. 5.

#### Comparison with relevant a-priori networks

Finally, we tested whether two brain networks, the Multiple Demand Network and the Theory of Mind Network, were involved in our task. As mentioned in Introduction, we expected to observe an involvement of the MDN as this network is associated with the representation of the rules and the facts relevant for deciding a course of action. This network should then be more active when the strategic environment is more complex and when it changes. Activation of the MDN during critical task phases should be associated with a better overall performance. By contrast, we did not expect to observe the involvement of the ToM network because of the absence of human counterparts (see Introduction). We used an analysis approach similar to the one employed for whole-brain analyses. For each of the two networks and for each ROI, we assessed whether the brain response to the strategic complexity followed our model’s predictions. Results are summarized in Fig. 3. Three quarters of the ROIs within the MDN (i.e., 12 out of 17, see Fig. 3, panel C) displayed an activation pattern consistent with that predicted by our model ($p < 0.05$, corrected for multiple comparisons). The same was true when we tested the activation pattern over the whole network ($p < 0.001$). A substantial proportion of the ROIs showing a correlation with the complexity index, also showed an interaction between our complexity measure and the Raven group (6 out of 13 ROIs), however only when we did not correct for multiple comparisons ($n = 13$). With this correction, none of the ROIs showed an effect, since the average *p* value was weak ($p=0.022$). The interaction was significant when the whole network was considered ($p = 0.030$). Finally, a large fraction of the MDN ROIs showed higher activation when the strategic environment changed as compared to when it did not change: out of the 17 MDN ROIs we considered, 14 showed higher activation during change trials ($p<0.05$, corrected for multiple comparisons). Overall, the results of the analysis on the MDN network closely mirrored those obtained in the whole-brain analyses. By contrast, none of the four ROIs usually associated with ToM that we considered, or the ROI set as a whole, displayed a significant effect of complexity. No effect was found also when we tested for a higher activation when the environment changed. To assess differences in the involvement of the MDN and the ToM network in our task, we directly compared the average neural response in the two networks across levels of complexity. We found a significant main effect of network ($\chi ^2(1)=1118.7, p<2.2 \times 10^{-16}$), that is, the mean activation in the two networks was significantly different. In particular, activation within the MDN was higher as compared to the ToM network. Furthermore, and importantly, we found a main effect of complexity ($\chi ^2(3)=174.1, p<2.2 \times 10^{-16}$) showing a correlation between brain activation and complexity, and an interaction effect between complexity and network ($\chi ^2(3)=35.8, p=8.4 \times 10^{-8}$) indicating a different response to complexity in the two networks.

## Discussion

As animals living in complex societies, humans often deal with situations where the outcome is determined not only by the combination of individuals’ actions and Nature but also by other people’s actions. Unlike Nature, other agents have memory and expectations, which may change how they react to repetitive encounters with the same individual. Multiple factors influence social (or strategic) decision-making. Previous research has mostly focused on emotional responses (e.g., disappointment, regret^60–64^), social reactions (e.g., envy, reciprocity^65–67^), or considerations of normative or moral nature^68–70^. This work addresses a less investigated factor: the role of cognition and its inter-individual variability in strategic decision-making. We first developed a new model of strategic decision-making in stochastic games. Then, we tested some empirical predictions of the model on both behavioral and neural data. The proposed model allows a measure of complexity in a game where participants understand others’ behavior and the stochastic nature of the environment, elaborate the best response, and implement this response correctly. The model also provides a measure of performance, which is obtained by comparing the optimal value in a state and the value obtained with the participant’s choice. This comparison provides the magnitude of the loss incurred when choosing the wrong action. Thus, we could evaluate how complexity, together with cognitive skills, would predict participants’ performance in each realization of the environment. Similarly, the model also provides expectations on patterns of brain activation associated with each environment. Our model of strategic interaction develops a game-theoretic analysis of strategies as automata^71,72^, as well as game-theoretic models of limited memory^73^, and is thus related to an important stream of research in Game Theory. Our study adds to these earlier contributions an experimental test and a link of the model to biological evidence.

In Introduction, we formulated three main predictions about behavior: (1) our measure of complexity would predict performance; (2) participants with lower cognitive ability would disproportionally fail in the more complex environments; and finally, (3) effects associated with non-cognitive traits would not emerge in our experiment.

Overall, our results provide support for these hypotheses. The average participants’ outcome (as measured by loss) was worse in more complex environments. For example, it was worse in *BoS* than *PD* and in *TfT* than *AD*. Furthermore, as expected, with the increasing complexity of the environment, the performance of participants with a lower fluid intelligence degraded at a higher rate than the performance of those with higher intelligence. Thus, even if all groups displayed a similar loss in the simplest strategic environment (i.e., *AD*), they were far apart in the most complex ones (e.g., *TfT* in *BoS*). Interestingly, even though the group with the lowest fluid intelligence produced more errors than the group with the highest fluid intelligence, they took a similar amount of time to make a choice, as shown by the similar average RTs. The average RTs of participants in the low intelligence group are even faster than those of participants with medium fluid intelligence. This pattern of loss and RTs in the three fluid intelligence groups (fast & good, slow & good, fast & bad for high, medium, and low intelligence groups, respectively) suggests that the participants with the lowest intelligence score adopted a qualitatively different strategy compared to the others. They likely reduced the complexity of the strategic environment at the expense of the outcomes attainable. Finally, we found no association between personality traits and performance indices, even for those traits (such as conscientiousness) that may have a beneficial role in cognitively demanding tasks.

Our neuroimaging results were consistent with our theoretical expectations and behavioral findings and provided insights into the brain processes underlying participants’ choices. First, neural activation correlated with the complexity of the strategic environment, being higher when the environment was more complex. Second, brain activation was also higher when the strategic environment changed, that is, when the participants had to reassess the strategic situation, as either the game or the partner changed. Finally, the neural activation of participants in the group with the lowest fluid intelligence was not modulated by the strategic complexity. Again, this observation hints at a qualitative change in how they processed the task compared to the participants with higher fluid intelligence.

Importantly, the effects consistent with our theoretical expectations were all observed within the fronto-parietal control system (or Multiple Demand Network^54^), a network involved in dynamically organizing and integrating the rules and the facts relevant for the decision flow^24–32,74^. By contrast, the Theory of Mind network, important for making inferences about others’ mental states^38–40^, was not involved. These findings imply that a network typically involved in single-agent tasks also has a central role in dealing with the cognitive aspects of interactive decision-making^75^: selecting relevant information, retaining it in working memory, integrating evidence from multiple interacting agents, and generating, evaluating, and applying an appropriate strategy. This conclusion is consistent with research assessing the economic aspects of social decision-making, showing that the same brain structures seem to encode those factors in social and non-social situations (for a review, see^76^). It will be important to confirm such observations in a task involving real, and not simulated, human partners.

The observed central role of the MDN may provide the neural pathway of the reported association between strategic sophistication and fluid intelligence^5^. On the one hand, we showed here that the MDN is involved in processing the cognitive aspects of strategic decision-making, particularly in groups with a higher Raven score. On the other hand, it has been shown that regions within the MDN are pivotal in determining inter-individual variations of fluid intelligence^35^.

In our analyses, we used time-resolved *f*MRI analysis to explore two task phases: the cue phase (i.e., the moment when participants receive information about the current game and partner) and the feedback phase (i.e., the time when participants are informed about the choice of their partner). We expected to find a similar pattern of neuroimaging results in the two stages. The effects of the complexity of the environment emerged more clearly in the feedback than in the cue phase. This suggests that, contrary to our expectations, a primary part of the integration of evidence and decision process took place at feedback. In a coordination game, the information about the opponent’s first move is decisive. The decision strategy of the most skilled participants may have extensively relied on the knowledge of the other player’s choice, mainly when the partner changed. Given that the choice information is only available at feedback, this strategy explains why participants would postpone most of the processing load to the feedback phase of the first trial upon game change.

The present study featured a relatively large number of participants (n = 42). This sample size grants an adequate statistical power for exploring even medium to small effect sizes in within-subject analyses (e.g., a power of 0.95 for an effect size with Cohen *F* = 0.15). However, this is not the case for between-subject analyses where power reduces to 0.59 even for large main effects of group factor. Thus, this study provides limited evidence on the real presence of small to medium effects of between-subject factors, such as, for example, personality factors. A further limitation is related to the population from which our participants were sampled. As often in neuroscience studies, participants were recruited on a university campus. The Raven score of university students is usually higher than that of the general population. Because of that, participants with very low Raven scores were underrepresented in our sample, thus producing a restriction of range in correlation estimations. However, we believe that the inclusion of low Raven participants would have made the observed relations between complexity in strategic interaction and fluid intelligence, if anything, stronger.

Together with previous works^3–5,77^ our findings strengthen the importance of cognitive factors in shaping social decision-making. This may also have implications for public policy if we think these policies should contribute to fostering a well-functioning society. Until now, great emphasis has been given to shaping social norms and emotional attitudes for social cohesion and much less to the cognitive aspects of cooperation. Our results indicate that this cognitive side of education may be just as important, even if the intent is to foster good citizenship. It is possible that the best avenue to educate good citizens is to nurture educated, far-sighted people.

## Supplementary Information

Supplementary Information.

## Data Availability

All analyses were performed using in-house developed code implemented in MATLAB and Stata. The scripts used for all the analyses and those that simulate the automata can be openly accessed at https://osf.io/h4n5c/. The *f*MRI data used to perform the analyses may be made available by the corresponding author (carlo.reverberi@unimib.it) upon request.
