# Supplementary material for: Strategic complexity and cognitive skills affect brain response in interactive decision-making
Source: Sci Rep. 2022 Sep 23;12:15896. doi: 10.1038/s41598-022-17951-0 (PMC9508177; doi:10.1038/s41598-022-17951-0)
Supplement: Supplementary file 1 — Supplementary Information. [file 41598_2022_17951_MOESM1_ESM.pdf]

Strategic Complexity and Cognitive Skills affect Brain Response in Interactive Decision-Making

– Supplementary Information –

Carlo Reverberi, Doris Pischedda, Marco Mantovani, John-Dylan Haynes, Aldo Rustichini

A Supplementary Analysis and Results

A.1 Raven scores and subgroups

Participants performed the Raven's Advanced Progressive Matrices, which is an appropriate test for our population of young adults of above-average intelligence. In particular, we administered the 20-minute version of the test, following the norms described in a previous work[44]. This version features the 36 items of Set II of the Advanced Progressive Matrices. Rather than the standard paper-and-pencil version, the test was computerized and run at the beginning of the training session, which preceded the *f*MRI session. In both the behavioral and the neuroimaging analysis, we split our sample into three subgroups, using as cutoff the 33 and 66 percentile of the distribution of the Raven scores. This distribution, as well as the cutoffs, are represented in Fig. A1.

Figure A1. Distribution of Raven Scores. The figure shows the violin plot of the distribution of Raven scores, indicating the median (white dot), the interquartile range (box), and the Gaussian Kernel density. The superimposed red horizontal lines indicate the 33 and 66 percentile cutoffs that distinguished the Low, Medium, and High Raven subgroups we used in the analysis.

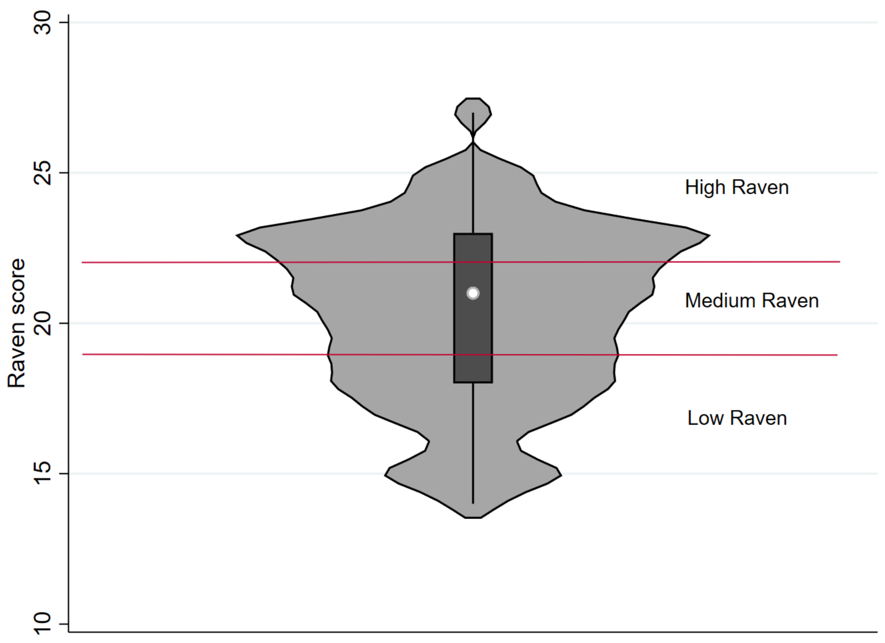

This split is motivated by the likely non-linear nature of the relation between the Raven score and the dependent variables. Figure A2 provides evidence of such a non-linear relation.

Figure A3 shows that our main results on the interaction between complexity and intelligence on loss and reaction times hold unchanged when we use the Raven score as a continuous variable and allow for non-linearities in the form of a quadratic

Figure A2. **Raven scores, loss and response times**. Marginal effects of Raven scores (used as continuous) on loss and RTs from mixed-effect models. These include both a linear and a quadratic term for the Raven score, as well as controls for the current trial and for whether the game or partner changes in the current trial.

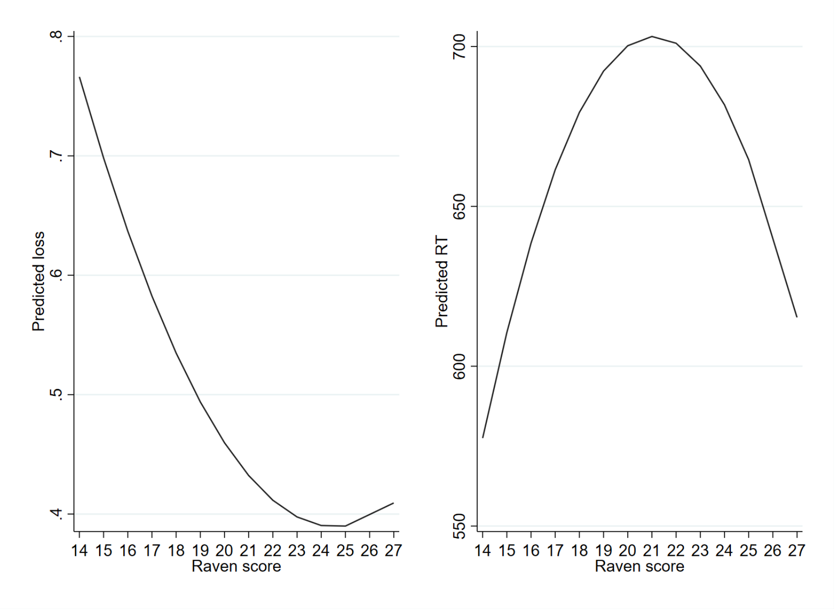

term for the Raven score: complexity affects more the performance of individuals with lower Raven scores, and individuals with medium Raven scores have a similar performance as individuals with higher scores but have the highest RTs of all.

Figure A3. **Interaction between complexity and Raven scores**. Marginal effects of Raven scores (used as continuous) on loss and RT at different levels of complexity. The effects are derived from mixed-effect models. These include both a linear and a quadratic term for the Raven score, as well as controls for the current trial and for whether the game or partner changes in the current trial.

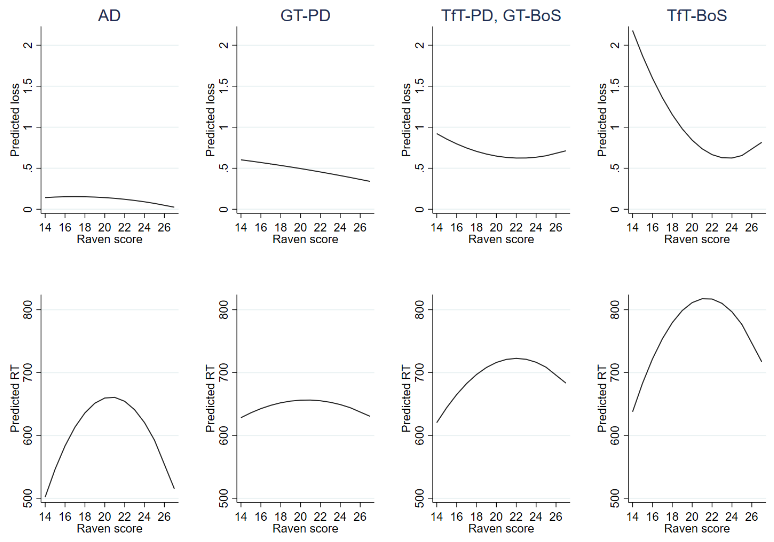

Table A1. Behavioral analyses: mixed models on the measure of complexity. The table reports a set of mixed models with random individual effects. The dependent variable is either the expected loss (odd columns) or the RT (even columns) in a given trial. 'Complexity' is the index of complexity based on pairwise comparisons of the transition matrices. 'Trial' is a continuous counter for the trial index. 'Switch' is a dummy variable that takes value 1 if the partner or the game changes in the current trial. 'Medium IQ' and 'Top IQ' indicate belonging to the intermediate or top third of the sample in terms of Raven score, respectively (i.e., the bottom third is the baseline category). *,**,***: statistically significant at the 10%, 5% and 1% level, respectively. Between parentheses, we report robust standard errors, clustered at the subject level to account for non-independence of observations from the same subject.

	(1)	(2)	(3)	(4)	(5)	(6)	(7)	(8)
	Whole sample		Switch trials		Stay trials		Whole sample	
	loss	RT	loss	RT	loss	RT	loss	RT
complexity	0.093***	17.207***	0.119***	29.415***	0.073***	7.209***	0.140***	16.707***
	(0.011)	(2.214)	(0.012)	(3.547)	(0.012)	(1.606)	(0.019)	(3.342)
trial	-0.000	-0.401***	0.000	-0.462***	-0.000	-0.316***	-0.000	-0.401***
	(0.000)	(0.075)	(0.000)	(0.108)	(0.000)	(0.071)	(0.000)	(0.075)
switch	0.217***	167.659***					0.217***	167.659***
	(0.038)	(19.487)					(0.038)	(19.490)
Medium IQ							-0.273**	111.796*
							(0.108)	(60.207)
Top IQ							-0.219**	16.358
							(0.106)	(56.772)
Medium IQ*complexity							-0.080***	-0.489
							(0.025)	(4.820)
Top IQ*complexity							-0.060**	1.990
							(0.024)	(5.293)
_cons	0.422***	662.652***	0.634***	845.567***	0.425***	649.409***	0.586***	619.934***
	(0.021)	(13.333)	(0.029)	(15.890)	(0.033)	(10.145)	(0.084)	(45.295)
N	12096	12096	5712	5712	6384	6384	12096	12096

A.2 Behavioral regressions

The behavioral analysis is based on mixed-effect models, where the main independent variable is the measure of complexity described in the Methods section. The independent variables are the loss variable, also derived from the conceptual framework in the Methods section, and reaction times. All the tests reported in the text, as well as the marginal effects shown in Fig. 4, are derived from the regressions reported in Table A1.

A.3 Identification of the critical time windows

With this accessory analysis, we aimed to identify which time bins correspond to the time window of interest in the task, namely the window spanning from cue to feedback presentation, including their processing. We know that a hemodynamic delay exists between the timing of real-world events and the hemodynamic response that they produce. This delay is usually in the range of two to four seconds. We relied on the brain response to motor acts to estimate the delay in our setting. Given the relatively fast RTs we observed in the task, evidence of motor activation is a reasonable proxy to detect the beginning of the cue phase. As a first-level analysis, we run an FIR model considering only button presses. We set the temporal parameters of the FIR model to cover the duration of a single trial, from the presentation of the cue to the post-feedback elaboration. The moment of cue presentation was used as the onset time for all events. Each FIR time bin lasted one TR (i.e., 2s), and each event was modeled with nine time-bins, thus covering 18s from the onset time. The FIR model included two conditions: left button press and right button press. At the second level, we compared activation related to left versus right button presses in each time bin. In particular, we looked for the earliest time bin where a significant difference was detectable. We applied a statistical threshold of $p < .001$ at the voxel-level uncorrected for multiple comparisons, and of $p < .05$ at the cluster level, family-wise error corrected for multiple comparisons[52]. As reported in Fig. A4, a difference in motor activation could be detected as early as in

the second time bin, reaching its peak at the third time bin. Given this evidence, we can safely conclude that cue presentation corresponds most likely to the second time bin.

Figure A4. **Temporal Unfolding of the Motor Activation**. Brain regions showing a motor effect, that is, the regions in which the activation associated with the left button press was different from that associated with the right button press (Panel A). The earliest time at which the motor effect was detectable was the second time bin, that is, from two to four seconds after onset, without considering the hemodynamic delay. The areas in the brain map are color-coded according to the color scale shown below, representing F-values. Clusters with significant activation at a threshold of 0.001 at the voxel level and of 0.05 at the cluster level (FWE corrected for multiple comparisons) are displayed. The temporal unfolding of the activation over the nine time-bins is considered for right (panel B) and left (panel C) button presses. Time bin one starts at cue onset. Each time bin lasts two seconds.

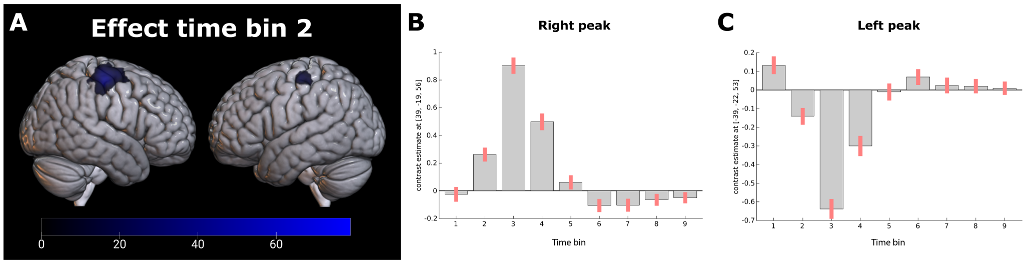

A.4 Change in the strategic environment.

As mentioned in the main text, to assess the robustness of our findings, we additionally employed an alternative approach to uncover brain regions involved in our task. We assumed that the critical cognitive processes under investigation are more taxed upon change in the strategic environment. Thus, we compared the brain activation between change and no-change trials, averaging across complexity levels. This analysis is statistically independent of the primary analysis on complexity effects. The results are displayed in Fig. A5. The observed fronto-parietal network is very similar to the one reported in Fig. 5, including areas showing an effect of complexity.

Figure A5. **Change of the strategic environment.** Brain regions showing a higher activation when the strategic environment changes, that is when either the partner or the game are different from the previous trial. The areas in the brain maps are color-coded according to the t-value scale shown at the bottom. The significant results are displayed using a threshold of 0.001 at the voxel level and of 0.05 at the cluster level (FWE corrected for multiple comparisons).

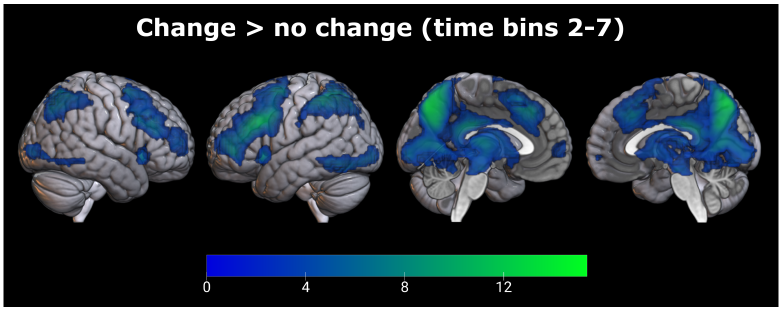

A.5 *f*MRI statistical tables

We report below (Tables A2-A4) the statistical tables of the main neuroimaging results described in the paper.

Table A2. Brain regions where the neural activation correlated with strategic complexity. The statistic table refers to the analysis over the whole time window considered, that is, considering both cue and feedback (see Fig. 5, Panel A). For each cluster, as defined within SPM, we report the corrected (FWE) p-value, the total number of voxels in the cluster, the t-value of cluster's peaks and their stereotactic coordinates in the MNI space.

cluster p	cluster size	peak t	x	y	z
<.001	2871	14.77	30	23	-4
		12.97	45	26	38
		12.6	6	35	38
<.001	2842	14.26	42	-58	47
		12.69	-42	-55	47
		12.14	39	-49	47
<.001	1572	13.98	-30	20	-4
		11.46	-36	47	-1
		11.42	-42	26	29
<.001	1113	9.34	9	-10	8
		8.53	12	14	-1
		7.57	-9	-10	5
0.001	159	7.33	-15	-52	-19
		6.22	3	-49	-16
0.001	157	6.28	3	-28	32
		4.73	-3	-13	29
0.016	86	5.97	63	-37	-1
		3.89	63	-49	-7

Table A3. Brain regions where the neural response to strategic complexity differed across Raven subgroups. This statistic table refers to the results reported in Fig. 6, Panel A. For each cluster, as defined within SPM, we report the corrected (FWE) *p*-value, the total number of voxels in the cluster, the F-value of cluster's peaks and their stereotactic coordinates in the MNI space.

**cluster *p***	**cluster size**	**peak F**	**x**	**y**	**z**
<0.001	606	39.05	-39	-67	44
		34.02	-39	-52	32
		20.56	-3	-61	41
<0.001	689	27.25	42	38	14
		24.52	33	14	35
		19.66	42	17	38
<0.001	164	27	45	50	2
		18.59	51	38	-10
		17.34	36	35	-1
<0.001	183	24.88	-36	50	-1
		15.16	-45	41	-10
		9.73	-48	38	17
<0.001	140	22.96	6	-16	-7
		15.25	15	-7	-4
		14.46	-6	-22	-7
<0.001	395	21.8	-39	17	23
		20.26	-36	20	32
		19.7	-30	5	65
<0.001	149	21.44	3	38	41
		15.7	-9	38	29
		9.19	6	47	32
<0.001	252	19.55	45	-61	50
		17.81	45	-40	35
		15.53	45	-64	35
0.033	55	17.74	0	-31	35
		10.67	-3	-40	26

Table A4. Brain regions responding to any change of the strategic environment. Changes could be due to the fact that either the partner or the game changed. This statistic table refers to the results reported in Fig. A5. We report the corrected (FWE) p-value for each maximum within the large single cluster, the total number of voxels in the cluster, the T-value of cluster's peaks and their stereotactic coordinates in the MNI space.

**cluster *p***	**cluster size**	**peak *t***	**x**	**y**	**z**
<0.001	16746	15.41	-33	-55	44
		14.97	-30	-64	41
		14.14	6	-70	50
		13.42	-6	-73	44
		13.42	-3	-70	50
		13.39	-9	-76	47
		12.95	-45	23	29
		12.87	-9	-70	59
		12.3	-39	29	26
		12.24	-45	-43	50
		12.09	-42	5	32
		11.86	30	-64	47
		11.62	-3	17	50
		11.28	15	-61	32
		11.18	12	-64	35
		11.13	-3	-31	26

B Transition matrices

In this section of the Supplementary Information, we report the transition matrices T_a and the utility $u(s,a)$ used for the computation of the value function, in the order

$$(PC, BC, PA, BA).$$

Here P stands for PD, C for cooperative, B for BoS, and A for aggressive. In the caption of each table, we recall the meaning of the action (for example: $A \sim d$ in PD indicates that the action A represents defection, namely Def, in the PD).

B.1 Transition matrices and utility for *AD, GT*

Tables B1-B4 are the four transition matrices for AD and GT. The names of the matrices are meant to represent the content. Each matrix identifies a game-action pair $\in \{PD, BoS\} \times \{C, A\}$ and each cell shows the probability of the transition from the row to the column internal state, given the game, opponent and the participant's action identified by the matrix name. For example, **AG-PC** represents the transition matrix for the AD and GT automata (hence **AG**) in the PD (**P**), when the player chooses cooperation (**C**); **AG-BC** represents the same situation, but for the BoS (hence, the **B**), and so on. The corresponding four utility vectors for the human participant follow in Tables B5-B8. As with the transition matrices, each vector identifies a game-action pair $\in \{PD, BoS\} \times \{C, A\}$. Each cell contains the payoff of the participant in the internal state identified by the row, given the game, opponent and participant's action identified by the vector name.

Table B1. $(g,t) = (P, ADGT), a = C \sim c.$

**AG − PC**	ADg'	$GTg'0b$	$GTg'0w$	$GTg'1b$	$GTg'1w$
ADP	1	0	0	0	0
$GTP0b$	0	1	0	0	0
$GTP0w$	0	0	1	0	0
$GTP1b$	0	0	0	1	0
$GTP1w$	0	0	0	0	1

Table B2. $(g,t) = (B, ADGT), a = C \sim w.$

**AG − BC**	ADg'	$GTg'0b$	$GTg'0w$	$GTg'1b$	$GTg'1w$
ADB	1	0	0	0	0
$GTB0b$	0	0	0	0	1
$GTB0w$	0	1	0	0	0
$GTB1b$	0	0	0	0	1
$GTB1w$	1	0	0	0	0

Table B3. $(g,t) = (P, ADGT), a = A \sim d.$

**AG − PA**	ADg'	$GTg'0b$	$GTg'0w$	$GTg'1b$	$GTg'1w$
ADP	1	0	0	0	0
$GTP0b$	1	0	0	0	0
$GTP0w$	1	0	0	0	0
$GTP1b$	1	0	0	0	0
$GTP1w$	1	0	0	0	0

Table B4. $(g,t) = (B, ADGT), a = A \sim b.$

$\mathbf{AG-BA}$	ADg'	$GTg'0b$	$GTg'0w$	$GTg'1b$	$GTg'1w$
ADB	1	0	0	0	0
$GTB0b$	0	0	1	0	0
$GTB0w$	0	0	0	1	0
$GTB1b$	1	0	0	0	0
$GTB1w$	0	0	0	1	0

Table B5. $(g,t) = (P, ADGT), a = C \sim c.$

$\mathbf{u}(\mathbf{AG-PC})$	ADg'
ADP	12
$GTP0b$	48
$GTP0w$	48
$GTP1b$	48
$GTP1w$	48

Table B6. $(g,t) = (B, ADGT), a = C \sim w.$

$\mathbf{u}(\mathbf{AG-BC})$	ADg'
ADB	25
$GTB0b$	25
$GTB0w$	0
$GTB1b$	25
$GTB1w$	0

Table B7. $(g,t) = (P, ADGT), a = A \sim d.$

$\mathbf{u}(\mathbf{AG-PA})$	ADg'
ADP	25
$GTP0b$	50
$GTP0w$	50
$GTP1b$	50
$GTP1w$	50

Table B8. $(g,t) = (B, ADGT), a = A \sim b.$

$\mathbf{u}(\mathbf{AG-BA})$	ADg'
ADB	0
$GTB0b$	0
$GTB0w$	48
$GTB1b$	0
$GTB1w$	48

B.2 Transition matrices and utility for *TfT*

Tables B9-B12 are the four transition matrices for TfT. As in B.1, each matrix identifies a game-action pair $\in \{PD, BoS\} \times \{C, A\}$ and each cell shows the probability of the transition from the row to the column internal state, given the game, opponent and the participant's action identified by the matrix name. The corresponding four utility vectors for the human participant follow in Tables B13-B16. As with the transition matrices, each vector identifies a game-action pair $\in \{PD, BoS\} \times \{C, A\}$. Each cell contains the payoff of the participant in the internal state identified by the row, given the game, opponent and participant's action identified by the vector name.

Table B9. $(g,t) = (P, TfT), a = C \sim c.$

**T − PC**	$Tg'00b$	$Tg'00w$	$Tg'01b$	$Tg'01w$	$Tg'10b$	$Tg'10w$	$Tg'11b$	$Tg'11w$
$TP00b$	1	0	0	0	0	0	0	0
$TP00w$	0	1	0	0	0	0	0	0
$TP01b$	1	0	0	0	0	0	0	0
$TP01w$	0	1	0	0	0	0	0	0
$TP10b$	0	0	0	0	1	0	0	0
$TP10w$	0	0	0	0	0	1	0	0
$TP11b$	0	0	0	0	1	0	0	0
$TP11w$	0	0	0	0	0	1	0	0

Table B10. $(g,t) = (B, TfT), a = C \sim w.$

**T − BC**	$Tg'00b$	$Tg'00w$	$Tg'01b$	$Tg'01w$	$Tg'10b$	$Tg'10w$	$Tg'11b$	$Tg'11w$
$TB00b$	0	0	0	0	0	1	0	0
$TB00w$	1	0	0	0	0	0	0	0
$TB01b$	0	0	0	0	0	1	0	0
$TB01w$	0	0	0	0	0	1	0	0
$TB10b$	0	0	0	0	0	1	0	0
$TB10w$	0	0	1	0	0	0	0	0
$TB11b$	0	0	0	0	0	1	0	0
$TB11w$	0	0	0	0	0	1	0	0

Table B11. $(g,t) = (P, TfT), a = A \sim d.$

**T − PA**	$Tg'00b$	$Tg'00w$	$Tg'01b$	$Tg'01w$	$Tg'10b$	$Tg'10w$	$Tg'11b$	$Tg'11w$
$TP00b$	0	0	1	0	0	0	0	0
$TP00w$	0	0	0	1	0	0	0	0
$TP01b$	0	0	1	0	0	0	0	0
$TP01w$	0	0	0	1	0	0	0	0
$TP10b$	0	0	0	0	0	0	1	0
$TP10w$	0	0	0	0	0	0	0	1
$TP11b$	0	0	0	0	0	0	1	0
$TP11w$	0	0	0	0	0	0	0	1

Table B12. $(g,t) = (B, TfT), a = A \sim b$

$\mathbf{T-BA}$	$Tg^{'}00b$	$Tg^{'}00w$	$Tg^{'}01b$	$Tg^{'}01w$	$Tg^{'}10b$	$Tg^{'}10w$	$Tg^{'}11b$	$Tg^{'}11w$
$TB00b$	0	1	0	0	0	0	0	0
$TB00w$	0	0	0	0	1	0	0	0
$TB01b$	0	1	0	0	0	0	0	0
$TB01w$	0	1	0	0	0	0	0	0
$TB10b$	0	0	0	1	0	0	0	0
$TB10w$	0	0	0	0	1	0	0	0
$TB11b$	0	1	0	0	0	0	0	0
$TB11w$	0	1	0	0	0	0	0	0

Table B13. $(g,t) = (P, TfT), a = C \sim c.$

$\mathbf{u(T-PC)}$	$Util$
$TP00b$	48
$TP00w$	48
$TP01b$	12
$TP01w$	12
$TP10b$	48
$TP10w$	48
$TP11b$	12
$TP11w$	12

Table B14. $(g,t) = (B, TfT), a = C \sim w$

$\mathbf{u(T-BC)}$	$Util$
$TB00b$	25
$TB00w$	0
$TB01b$	25
$TB01w$	25
$TB10b$	25
$TB10w$	0
$TB11b$	25
$TB11w$	25

Table B15. $(g,t) = (P, TfT), a = A \sim d.$

$\mathbf{u(T-PA)}$	$Util$
$TP00b$	50
$TP00w$	50
$TP01b$	25
$TP01w$	25
$TP10b$	50
$TP10w$	50
$TP11b$	25
$TP11w$	25

Table B16. $(g,t) = (B, TfT), a = A \sim b$

$\mathbf{u(T-BA)}$	$Util$
$TB00b$	0
$TB00w$	48
$TB01b$	0
$TB01w$	0
$TB10b$	0
$TB10w$	48
$TB11b$	0
$TB11w$	0

B.3 Transition and utility vectors

Finally, we collect the different components to define the elements in the Bellman equation (2). The transition matrices appearing in Table B17 are defined in the previous sections.

Table B17. Matrix T_a, for $a \in \{C,A\}$. This is a $(5+5+8+8)^2$ matrix.

$\mathbf{T_a}$	$AG-P$	$AG-B$	$T-P$	$T-B$
$AG-P$	$\rho AG-Pa$	$(1-\rho)AG-Pa$	$0_{5\times 8}$	$0_{5\times 8}$
$AG-B$	$(1-\rho)AG-Ba$	$\rho AG-Ba$	$0_{5\times 8}$	$0_{5\times 8}$
$T-P$	$0_{8\times 5}$	$0_{8\times 5}$	$\rho T-Pa$	$(1-\rho)T-Pa$
$T-B$	$0_{8\times 5}$	$0_{8\times 5}$	$(1-\rho)T-Ba$	$\rho T-Ba$

The operator T_a in the Bellman equation (2) takes with probability $1-\pi$ the inner product of the transpose of this vector with the vector ϕ. This vector is independent of the state, so this component of the transition matrix results from 26 copies of the same row vector.

B.3.1 Initial States

Table B18 describes the initial states. The important information in this case is that the GT and the AD automata both begin with the choice of b (i.e., the choice that potentially gives the best payoff to the player) in BoS, whereas the TfT automaton begins with the choice of w.

Table B18. Vector of probability in the initial states.

AP	$\frac{1}{6}$
$GP0b$	$\frac{1}{6}$
$GP0w$	0
$GP1b$	0
$GP1w$	0
AB	$\frac{1}{6}$
$GB0b$	$\frac{1}{6}$
$GB0w$	0
$GB1b$	0
$GB1w$	0
$TP00b$	0
$TP00w$	$\frac{1}{6}$
$TP01b$	0
$TP01w$	0
$TP10b$	0
$TP10w$	0
$TP11b$	0
$TP11w$	0
$TB00b$	0
$TB00w$	$\frac{1}{6}$
$TB01b$	0
$TB01w$	0
$TB10b$	0
$TB10w$	0
$TB11b$	0
$TB11w$	0

The final ingredient is the current utility $u(s,a)$, reported in Table B19 as two vectors of utility, one for each action.

Table B19. Utility for each state, depending on the action chosen by the subject.

	u if $a = C$	u if $a = A$
$AG - P$	$u(AG - PC)$	$u(AG - PA)$
$AG - B$	$u(AG - BC)$	$u(AG - BA)$
$T - P$	$u(T - PC)$	$u(T - PA)$
$T - B$	$u(T - BC)$	$u(T - BA)$
